# Referrals and Determinant Factors of a National School Health Campaign in Lebanon on Children Aged between 3 and 12 Years Old

**DOI:** 10.3390/children11020175

**Published:** 2024-01-30

**Authors:** Peter Habchy, Léa Tahan, Charbel Moussi, Muhammad A. Barakat, Laura Ghanem, Ogarite Kattan, Alain Njeim, Leila Abou Habib, Wassim El Bitar, Béchara El Asmar, Mirna N. Chahine

**Affiliations:** 1 Faculty of Medical Sciences, Lebanese University, Hadath 1519, Lebanon; p.habchy@st.ul.edu.lb (P.H.); lea.tahan@st.ul.edu.lb (L.T.); c.moussa@st.ul.edu.lb (C.M.); mohammad.barakat@st.ul.edu.lb (M.A.B.); laura.ghanem@st.ul.edu.lb (L.G.); o.kattan@st.ul.edu.lb (O.K.); a.njeim@st.ul.edu.lb (A.N.); 2 Lebanese Association of the Knights of Malta (Order of Malta Lebanon), Vanlian Bldg, 6th Fl. City Rama Str. Dekwaneh, Beirut P.O. Box 11-4286, Lebanon; leila.sebeel@yahoo.com (L.A.H.); wassim.elbitar@chirec.be (W.E.B.); bechara.elasmar@hdf.usj.edu.lb (B.E.A.); 3 Department of Pediatrics, Bellevue Medical Center University Hospital, Mansourieh P.O. Box 295, Lebanon; 4 Faculty of Medicine, Saint Joseph University, Beirut P.O. Box 17-5208, Lebanon; 5 Department of Cardiology, Hotel-Dieu de France Hospital, Achrafieh, Beirut 1100, Lebanon; 6 Basic Sciences Department, Faculty of Medical Sciences, Lebanese University, Hadath 1519, Lebanon; 7 Foundation-Medical Research Institutes (F-MRI^®^), Achrafieh, Beirut 1100, Lebanon; 8 Foundation-Medical Research Institutes (F-MRI^®^), 1211 Geneva, Switzerland

**Keywords:** children, Lebanon, health campaign, schools, interventions, screening, mental health

## Abstract

In this extensive study examining the health of 7184 school children aged 3 to 12 in 27 Lebanese schools, screenings involved medical evaluation and interviews, complemented by phone interviews with 3880 parents. Notably, one in two students received a medical referral, revealing prevalent issues such as dental cavities (33%), under-vaccination (25%), undetected vision problems (13%), abnormal growth (6%), underweight (27%), and overweight (33%). Additional concerns encompassed abnormal vital signs (3%), abuse signs (0.6%), infectious skin lesions (1.6%), scoliosis (1.7%), abnormal auscultation (heart 1.1%, lungs 1.2%), ear problems (3.3%), precocious puberty (0.7%), and neurologic signs (0.6%). Mental health challenges affected 20–25% of students. Public schools and Beirut exhibited higher referral rates, with girls, older children, overweight students, those lacking regular pediatrician visits, and children of self-employed or less educated parents facing elevated referral rates. In contrast, children of healthcare workers experienced fewer referrals. Against this backdrop, the study emphasizes the imperative for targeted health initiatives, particularly in marginalized areas and for socioeconomically disadvantaged students. Priority areas include dental care, weight issues, mental health, vaccination compliance, and addressing vision problems to enhance learning outcomes.

## 1. Introduction

### 1.1. Background

Survive, thrive, and transform—these are the three objectives adopted by the World Health Organization (WHO) in their global strategy for children’s health in order to end preventable death, ensure the health and well-being of children, and expand enabling environments, respectively [1]. These widely recognized aims can be promoted by a school-based health screening program since schools are then called to play a significant role in addressing social and health issues, given their strategic capacity to reach children and families [2,3].

In developing countries, due to inadequate health services for the general public and little understanding of healthcare, parents and teachers are unable to identify health issues in children that can disturb their learning ability, such as vision and hearing deficits. However, health screening in the form of medical check-ups carried out through schools and addressed at an early stage can identify learning difficulties or disorders in some children who can be saved from losing interest in their studies [4]. Similarly, regular screening of students for health conditions and communicable diseases by schools can detect and identify potential health risks early and enable appropriate measures to prevent the spread of infectious diseases [5,6].

We should also point out that children’s health is not restricted to physical health only since mental health is crucial in their overall well-being, as stated by the WHO definition of health [7]. Over the past decade, increasing rates of mental health issues among young people have been observed [8], and early childhood adversities, such as abuse or neglect, have been implicated in lasting consequences and health modifications [9]. Therefore, the first step in providing targeted support for children at risk and their caretakers could be screening for exposure to those childhood experiences, protective factors, and effects of a toxic stress response [9].

On a national level, despite all the unfortunate compounded economic and health crises that assailed and are still assailing Lebanon, the Ministry of Public Health (MoPH) did not fail to implement and improve numerous school health initiatives (medical screening, health awareness, and healthy school environment) and to pay close attention to preventive and restorative health programs targeting youth and their well-being within a whole school community [10].

However, it is important to mention that health-promoting schools collaborate and integrate efforts not only from students’ perspectives but also from parents’ perspectives in order to promote students’ health. This requires the commitment and participation of both the community and parents in expanding such programs [11]. Furthermore, despite having labs and medical equipment at their disposal, physicians often rely on parents’ observations and insights in Pediatrics [12]. Yet, the role of parental involvement was only tackled by an extremely limited number of adequate school health interventions [13].

### 1.2. Objectives

In this project held by the Order of Malta Lebanon (OML), an apolitical humanitarian organization whose core mission, among many, is the community health centers (OML medical centers and MMUs (Medical Mobile Units)) distributed all over Lebanon, we evaluated the health situation of children aged between 3 and 12 years in schools. The assessment involved two main components: (1) medical evaluation and health screenings on the children’s physical health and (2) face-to-face interviews on the children’s mental health. Additionally, the project involved phone call interviews with the parents of the school children to obtain supplementary information about the children’s medical conditions as well as their mental health. By collecting these data, the study aims to gain a comprehensive understanding of the health situation of school children and the various factors that may be impacting their health.

## 2. Materials and Methods

### 2.1. Participants and Study Design

**Figure 1:**
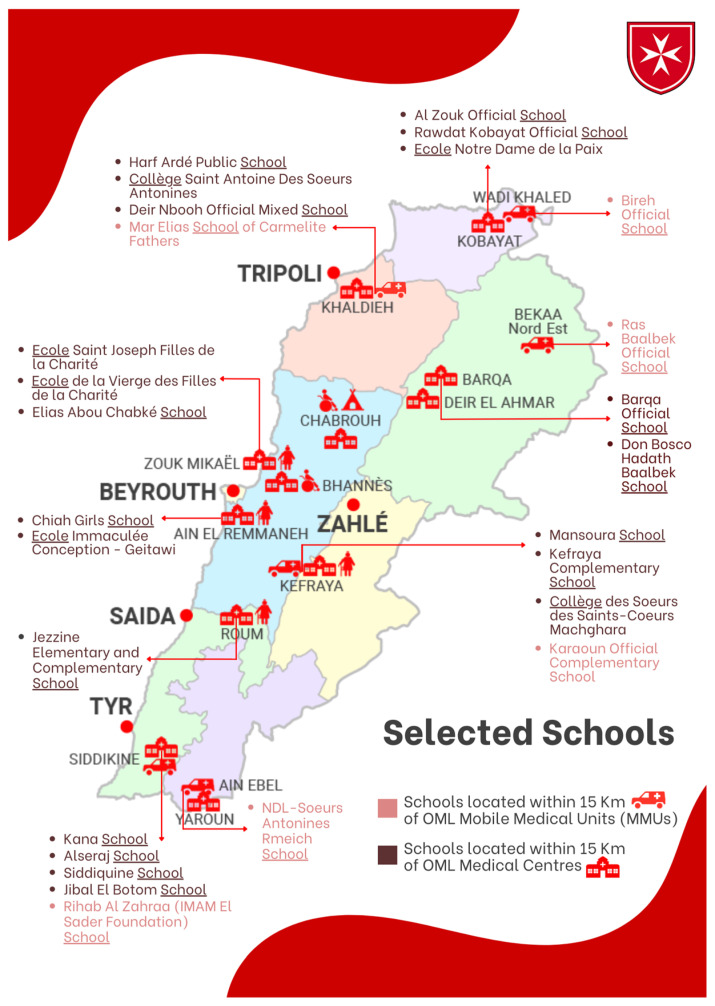
Selected schools in proximity of OML centers and MMUs. OML: Order of Malta Lebanon; MMUs: Mobile Medical Units.

A large cross-sectional study was carried out in 27 schools (16 public schools and 11 semi-private ones with variable sizes) widely distributed all over Lebanon, located at a perimeter of maximum of 15 km surrounding the OML medical centers and MMUs. For each OML medical center or MMU, around 2 to 3 public schools and 1 semi-private school were chosen (Figure 1). We included school children of any nationality between 3 and 12 years of age (from kindergarten to Grade 7). We excluded the school children in the above-mentioned grades who fell outside the specified age range.

**Figure 2:**
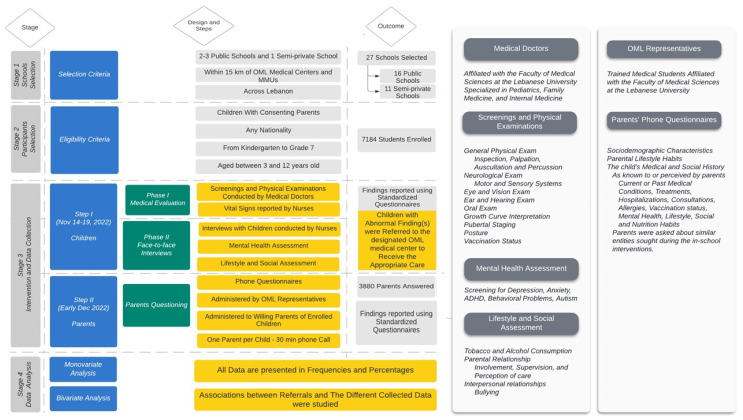
Study flow. OML: Order of Malta; MMUs: Mobile Medical Units; highlighted in yellow are the key elements directly related to the study objectives, serving as the main workflow of the research.

To examine the topic at hand, we organized our study into these two distinct steps (Figure 2):

The primary step consisted of a direct intervention that occurred in schools, which was divided into two phases. In the first phase, both medical doctors and nurses conducted a child’s medical evaluation and health screenings. In the second phase, nurses directly interviewed the child and asked about their mental health. Nurses and medical doctors performed these interventions privately in an appropriate environment for each child. They reported these findings for each child through standardized questionnaires 10 to 15 min in duration. This step took place between 14 and 19 November 2022.

The secondary step involved OML representatives (medical staff) conducting phone interviews with children’s parents using a questionnaire about any physical or mental condition the students may be experiencing. In addition, socio-demographic characteristics were assessed. The phone call took 30 to 35 min per parent. It was carried out within the first ten days of December 2022.

Besides the permission obtained from the Ministry of Education, this work was controlled by medical staff and executives from the OML (a nurse, a pediatrician, the head of the research department, and the head of the medical department at OML) involved in this campaign. Also, this research was set in collaboration with the faculty of medical sciences at Lebanese University.

### 2.2. Data Collection

A total of 68 medical doctors and 112 nurses were employed to conduct the fieldwork; most physicians are from the Faculty of Medical Sciences at the Lebanese University and are specialized in Pediatrics, Family Medicine, or Internal Medicine. Overall, 83 Lebanese university medical students were recruited as OML medical staff representatives to interview children’s parents and complete the medical file of each child. They were trained to verify the parents’ consent, to deliver a message of introduction provided by the OML communication department, and to ensure the good quality of their performance.

Data were collected by 3 separate questionnaires filled over Google Forms; first form was termed “Nurse findings” and included vital signs and face-to-face mental assessment interview performed and filled by the nurses. Second form was termed “Doctor findings” and included the screening and physical exam findings filled by the medical doctors during their auscultatory session with each child. Third form was termed “Parents’ questionnaire” and gathered the child’s medical history as well as their mental health; it was filled by the OML medical staff while asking the children’s parents the questions over the phone via WhatsApp calls.

“Nurse findings” and “Doctor findings” parameters were prepared by the OML nurse and the OML pediatrician. These parameters were further detailed and developed by the medical interns using several references [14,15,16]. “Parents’ questionnaire” was prepared according to a school health questionnaire by Dr. Flore Martini [17] and by the OML nurse. All three questionnaires were revised and edited by the research department. They were translated from English to French and Arabic languages using the inverted method of Fortin [18].

### 2.3. Variables

The medical evaluation variables cover vital signs, weight, height, growth curve percentile, general physical exam findings, eye exam findings, ear exam findings, oral exam findings, pubertal Tanner stage, posture, vaccination status, and referrals with abnormal findings. Interview variables include mental assessment and referral with mental health issues. Instruments of measurement were only used for variables including vision assessed with mini-Snellen eye chart, blood pressure measured with a pediatric cuff, height measured with an anthropometer, and weight measured with a weight scale.

Variables obtained from parents’ phone interviews include their children’s medical conditions, mental health, lifestyle habits, and nutrition habits. Physical health variables cover family medical history, past medical history, past surgical history, recent health issues, speaking and hearing problems, vision problems, learning difficulties, food allergies, medical care behavior, and vaccination status. Mental health variables involve mental disorders, relational and emotional life status, weight status, screen time, and sleep behavior.

### 2.4. Statistical Analysis

Data were analyzed using SPSS version 25.

Univariate analysis: Univariate analysis was enrolled, and all the study variables were presented. Nominal data (qualitative) were represented as frequencies and proportions. As for the continuous variables, results were presented by frequencies’ mean, standard deviation, and minimum and maximum values.

Referrals: All the referral data were presented by frequencies and proportions. Children were referred to designated OML medical center if they had abnormal vital signs, abnormal growth, an abnormal vision exam, a mental health problem, an atypical lesion (signs of abuse), infectious skin lesions, abnormal eye exam, abnormal posture, abnormal heart auscultation, abnormal lung auscultation, abnormal thyroid exam, an enlarged node or/and organ, an ear abnormality, an oral abnormality, an early pubertal stage, an abnormal neurological exam, or an incomplete vaccination.

All the referral data were tested and represented in the function of all the study variables (demographics of children, demographics of parents, school-related characteristics, comorbidities).

Tests used in the bivariate analysis were Chi-square test and Fisher exact test.

A statistically significant association was set at 5% (*p*-value less than 0.05).

### 2.5. Ethical Consideration

Before we started our study, under the supervision of Order of Malta Lebanon, we obtained approval from the Ministries of Public Health (MoPh)/Education (Approval number 3/10460 received on 5 October 2022). This study was conducted in accordance with Good Clinical Practice ICH Section Three and the principles laid down by the 18th World Medical Assembly (Helsinki, 1964 [19]) and all applicable amendments. Responses were confidential and were only used for research purposes. Parents and their children were asked by the school administration to electronically sign an informed consent in Arabic if they agreed to participate voluntarily in our study. In the informed consent, a detailed explanation of the background, objectives, risks, and advantages of the study was provided.

## 3. Results

For the purpose of this school health project and to better understand the health situation of children and the implicated factors, 7184 students all over Lebanon were subject to medical screenings. The sociodemographic characteristics of our study population are described in Table 1. This information was obtained during both the day of the campaign and from the 3880 parents in phone interviews.

In addition, further information regarding the medical history and the medical status (vaccination status, treatment, support device, hospitalizations, medical follow-up, hearing and vision status, and learning difficulties) of the children, as reported by the parents, is also represented in Table 2.

Furthermore, the findings of a mental status and social habits assessment of children are listed in Table 3. These findings were also obtained during both the day of the campaign and from the 3880 parents in phone interviews.

Referrals to the nearby OML medical centers were filed by medical doctors for abnormal findings. All types of medical (physical and mental assessments) referrals and their frequencies are listed in Table 4 and are described in the next paragraphs.

Regarding overall referrals, we found at least one referral-worthy finding in nearly half of our study participants (3508 students) (Table 4).

In the subsequent sections, we outlined the bivariate analysis conducted to highlight the associations between medical referrals and the implicated factors. For a more in-depth exploration of factors that were significantly correlated with referrals, additional detailed information is available in Supplementary Tables S1–S3.

The sociodemographic factors include school type, location, gender, nationality, age, parents’ occupation, and parents’ level of education. Our analysis revealed that public schools were more implicated in referrals than semi-private schools. Beirut, as a governorate, and Kefraya, as a nearby OML center, had higher percentages of referred children compared to other governorates and OML centers. Girls were more prone to referrals. Older children (10–12 years old) were more implicated in referrals. The children of parents working in healthcare had the lowest referral rates. Additionally, we found no significant variation in referral rates between children with employed or self-employed working mothers versus those with stay-at-home mothers. However, we observed a notable difference in referral rates among children based on their fathers’ employment status. Specifically, children of self-employed fathers had higher referral rates compared to those of fathers with other types of employment. Referral rates were decreasing with the increase in the mother’s level of education; that was not the case with fathers. Higher rates of referrals were observed at a middling education level (primary and complementary) and lower rates at both ends of the educational level spectrum (no education and higher level of education). Children with current smoker fathers showed lower referral rates than kids with non-current smoker fathers, and the highest level of referred children was among those with fathers who used to smoke. On the other hand, there were no significant findings regarding family income, parents’ marital status, and mother’s smoking status. However, results showed a significantly higher percentage of referred students among overweight children with higher BMI compared to the other BMI classes, probably due to the health disadvantages associated with obesity.

Regarding the other factors that were significantly correlated with referrals, students without a pediatrician had significantly more referrals than those who regularly visited a pediatrician at least once a year. However, there was no significant difference in overall referrals regarding medical history, ongoing treatment, or past psychologist visits.

In the following sections, we present the noteworthy and significant findings (*p* value < 0.05) that deviate from the overall referrals analysis.

Vital signs checkup: 218 (3%) students were referred for abnormal findings.

Growth status: 429 (5.9%) students were referred for abnormal growth, judged by the interpretation of the child’s weight and height growth curves. A noteworthy increase in abnormal growth referrals was observed in the area designated to Kefraya’s OML health center. Syrian showed higher referrals in this matter compared to Lebanese and other nationalities. Lower-income families had significantly more abnormal growth referrals than wealthier families. As for parents’ level of education, there was a higher rate of referrals in children with less educated mothers and fathers. Both underweight and overweight kids were more implicated in this referral compared to children with normal weight. Interestingly, we found significantly lower referrals related to abnormal growth in children undergoing a certain treatment.

Vision exam: 1050 (15.3%) students had a vision of anything less than 20/20 vision on the Snellen eye chart, and 955 (13.3%) were referred. The Bekaa and Baalbek-Hermel governorates showed significantly high abnormal vision referrals along with the Beirut governorate. In contrast to other referrals, abnormal vison was more reported in children with higher income families.

Skin inspection: the majority of examined students had normal skin despite 117 (1.6%) having pale skin, 5 (0.1%) having icteric skin, and 7 (0.1%) having cyanotic skin. There were 51 (0.7%) students with signs of hypercholesterolemia (yellowish skin deposits), and 43 (0.6%) students were referred for atypical skin lesions consistent with child abuse. We found higher rates of abuse referrals in children with retired fathers. Additionally, 118 (1.6%) children were referred for infectious skin lesions, such as lice, scabies, mycosis, impetigo, varicella, measles, and other infectious rashes, as represented in Table 4. Schools near Room OML medical centers were most implicated in the infectious skin lesion referrals. Children with positive medical histories showed fewer referrals in this matter.

Posture exam: 120 (1.7%) children were referred for abnormal posture, mostly from scoliosis. There were more referrals for abnormal posture in private schools than in public schools. Mount Lebanon governorate and schools near Zouk’s OML health center were most implicated.

Heart auscultation: 80 (1.1%) were referred for murmurs, extra heart sounds, and other abnormal heart auscultation. Schools designated to the OML Khaldieh Health Center had the highest referral rates.

Lungs auscultation: 83 (1.2%) were referred for decreased air entry and adventitious sounds. Younger children (3–6 years) had significantly higher referrals than older children. Schools in the Kobayat OML health center territory were most implicated. Unexpectedly, our data showed more abnormal lung auscultation in students with mothers who had higher levels of education.

Lymph nodes and organ palpation: 50 (0.7%) were referred for having hepatomegaly, splenomegaly, and adenopathy at different sites requiring further investigation. Bekaa and Baalbek-Hermel had significantly higher rates of this referral compared to other governorates. The same goes for children with lower family incomes.

Ear exams: 312 (3.3%) were referred for having otitis, a hearing disorder, or other ear problems. Private schools had higher referral rates than public schools. Abnormal ear exams were noted more in younger children (3 to 6 years). Schools within the OML Ain-el-Remmaneh health center territory were more implicated in this referral. Children with positive medical histories showed lower referral rates.

Oral exams: 1259 (17.5%) were referred for oral abnormality, whether for dental cavities, aphthous ulcers, or tonsil infections that necessitate medical care. The Mount Lebanon governorate and schools in OML Kobayat health center territory were most implicated in this referral. Lower-income families had significantly higher referred children than wealthier families. Similar to the overall referrals, parents’ level of education was significantly involved in abnormal oral exam referrals.

Pubertal stage exam: 51 (0.7%) were referred for early pubertal signs seen in younger children with higher Tanner stage than expected for their age. Children aged between 7 and 9 years were most implicated. Contrary to other referrals, boys were found to be more likely to be referred for precocious puberty than girls. To our surprise, children with positive surgical history had higher rates of this referral, unlike any other referrals where no significant finding was found regarding surgical history.

Neurological exam: 45 (0.6%) were referred for neurologic findings. Schools in the OML Kobayat Health Center territory were most implicated. Our data also showed that children who visited a psychologist in the past were more likely to be referred for abnormal neurological exams.

Vaccination checkup: by inspecting the health booklets of students, we found that 1527 (23.3%) had delayed vaccination visits according to their age. Furthermore, 1670 (25.4%) were referred for incomplete vaccination, respecting the Lebanese MOPH guidelines. It is important to stress the fact that public schools had significantly higher incomplete vaccination referrals than private schools. Schools within the OML Room’s health center territory and lower-income families had the highest rates of incomplete vaccination referrals, whereas children of parents with higher levels of education and who work in healthcare have the lowest rates. Furthermore, children with a positive medical history were more likely to be referred for incomplete vaccination.

Mental health checkup: after a thorough face-to-face interview with the children, 120 (1.7%) were referred for mental health issues demanding further follow-up. The detailed results of the mental health questionnaire are listed in Table 3. A brief summary showed that 1714 (23.8%) of students were recently bullied, 2181 (30.3%) stated that their parents recently did not understand their worries, 1452 (20.2%) felt lonely, 1599 (22.2%) experienced worrying sleepless nights, and 1892 (26.3%) were sad or stressed in the past year. Mount Lebanon governorate and areas designated to OML Kobayat medical centers and MMUs had the highest mental referral rates. In contrast to what was anticipated, fewer children were referred for mental health issues if they had a positive medical history.

**Figure 3:**
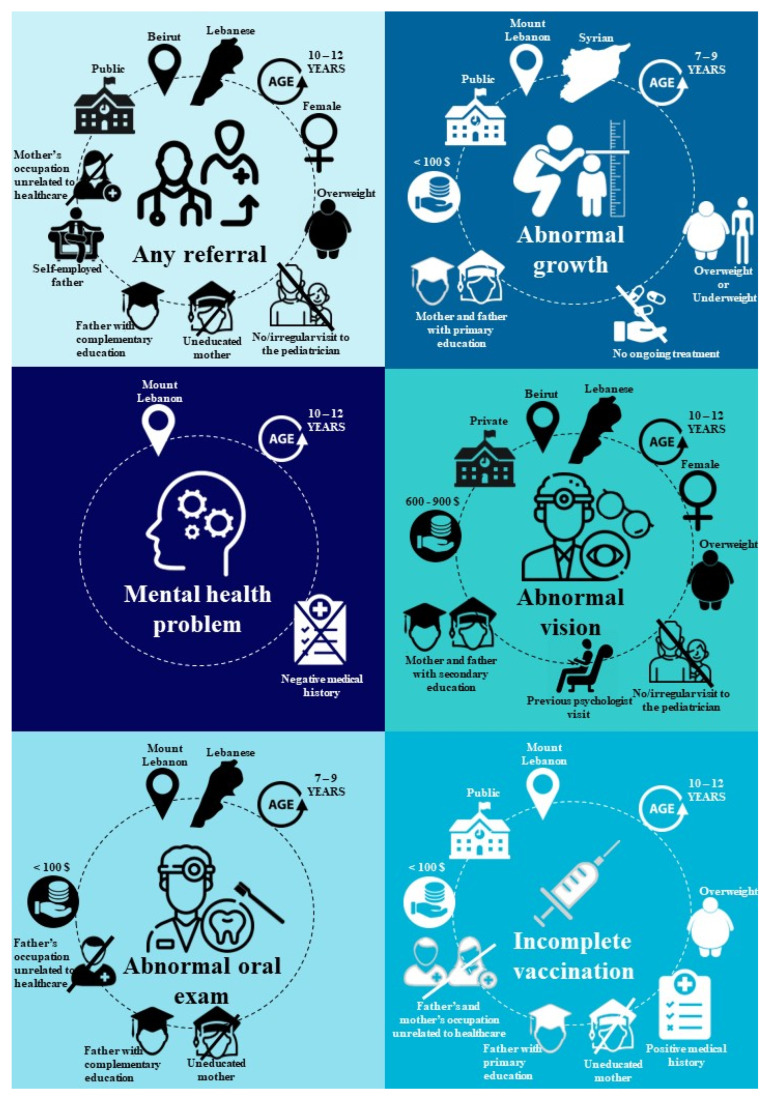
Factors significantly associated with the most prevalent types of referrals in the enrolled students.

Figure 3 summarizes the factors that are correlated with the most prevalent types of referrals in the study population.

## 4. Discussion

This study highlights findings regarding the health of school-aged children in Lebanon. Overall, our research showed that one in two students received one or more referrals for medical conditions that require further attention. Additionally, one in three children is found to have dental cavities, one in four did not complete their vaccination, 13% had undetected vision problems, and 6% were not within normal range of growth. Only 40% of the students have a normal weight based on their age-corresponding BMI, while 27% are underweight and 33% are overweight. Furthermore, urgent infectious cases, such as scabies and impetigo, were also detected and referred for immediate intervention. These alarming findings emphasize the current health situation of school-aged children in Lebanon and the need for urgent attention to address these health issues and ensure the well-being of the students.

### 4.1. Overall Referrals Findings

Our school health screening program resulted in a higher overall referral rate than previous school projects [20,21,22]. It also consisted of more modules of referral than any other screening program in the literature; the latter tends to focus on one or few aspects of screening like vision screening, heart screening, etc. One way to explain this difference in referral rates is the rise in poverty amidst the economic crisis in Lebanon and the subsequent health consequences it brings, which most certainly played a significant role in these high referral rates. United Nations International Children’s Emergency Fund (UNICEF) rapid assessments indicated that the percentage of children who were unable to receive necessary healthcare increased from 28% to 34% [23]. Additionally, another study from the United Nations Economic and Social Commission for Western Asia (UNESCWA) found that more than half of families were unable to acquire essential medications [24].

Moreover, our findings showed that students enrolled in public schools were more likely to be referred for abnormal health findings than those in private schools. This agrees with similar findings stated in the GSHS report regarding oral health, mental health, and physical activity [10]. Public schooling in Lebanon is not to be compared to public education in developed countries; it faces major problems, from poor-quality education to inadequate conditions for school operations [25]. Yet, it can be the only choice for underprivileged and marginalized students who are at higher risk of poor health, as many studies highlighted low socioeconomic status with unfavorable health outcomes [26].

Healthcare workers were shown to have a better clinical eye on their children despite their demanding professions; their children had the lowest referral rates compared to any other parents’ occupational status. To our knowledge, there is no evidence to back up this association, but we suggest that healthcare workers are often well-informed about health and wellness practices, which they may pass on to their children. However, this disagrees with a paper studying the psychological effects and health sequelae of healthcare workers’ children during the COVID-19 pandemic [27]. Mothers’ occupations did not affect the rate of referrals, indicating that working mothers, like stay-at-home mothers, were equally attentive to their children’s health needs and did not fail to seek medical attention when necessary. This contradicts the findings of some studies where children of working mothers have better health outcomes than those of non-working mothers [28], although there are still mixed results regarding maternal employment’s impact on children’s well-being [29]. As for fathers’ occupations, it was shown that the children of self-employed fathers had higher referral rates. Indeed, self-employed fathers tend to be more engaged in work than those with other types of employment [30], and those fathers probably lack attention to and concern about their children’s health.

Findings regarding parents’ education are well-represented in the literature [31]. Mothers’ education was more clearly implicated in children’s well-being compared to fathers’ education; the latter even had no significant effect in a comparative study conducted in Nepal [32].

Additionally, because of the increased health risks associated with obesity [33], it is understandable why this school screening, among others, flagged more children with higher BMI for abnormal findings and further evaluation.

Moreover, children who attend regular visits to a pediatrician were less likely to require referrals after our screening. Indeed, well-child visits play a critical role in the early detection and prevention of health problems, as well as tracking growth, providing immunizations, educating families about healthy practices, and decreasing in risks of preventable hospitalization [34].

### 4.2. Physical Health Assessments

#### 4.2.1. Vaccination Status

Three out of four of our students completed their recommended vaccines, aligning with a million children studied in the United States [35]. However, rates were much higher in less developed countries [36,37]. This confirms the high rates of vaccine compliance seen nationally with mandatory vaccines [38]. Nevertheless, overall vaccination rates dropped alarmingly in Lebanon since the COVID-19 pandemic [39], and interventions are needed to prevent further decline. In fact, five cases of measles (a vaccine-preventable disease) were identified in our school intervention.

Immunization practices were indeed influenced by the socioeconomic factors of the family. Actually, many international studies linked higher family income and higher levels of education with lower vaccination rates since those parents tend to question vaccine safety [40]. Our results also revealed a lack of compliance with recommended vaccinations. There are various factors that contribute to this disparity, including the lack of accessibility and affordability of vaccines since universal health coverage is not granted in Lebanon, in addition to the lack of knowledge about the benefits of vaccines and the severity of vaccine-preventable diseases [41].

#### 4.2.2. Oral Exam Findings

Oral health, a leading concern noted in our findings, revealed dental cavities in 35% of our students. In fact, our rate is mildly lower than the world prevalence [22,42], yet it is still a significant cause of concern.

The reason behind its association with low socioeconomic status must be the lack of access to dental care and oral health education [43]. Furthermore, parents with higher education levels tend to have better oral health knowledge and imply better oral hygiene practices [44].

#### 4.2.3. Vision Exam Findings

In developed countries, school-based vision screenings were found to have lower referral rates compared to our study in Lebanon [45,46]. It is most likely due to the unfortunate economic constraints that limit access to eye care services in our population [47]. However, research shows a higher prevalence of asymptomatic vision abnormalities, especially in children of our population’s age [48]. These are alarming findings, considering that education in schools relies heavily on visual learning. Our findings also revealed a correlation between abnormal vision referrals and higher-income families, as seen in one study we found from Nigeria [49] but contradicted by many papers [50]. While it may be speculative, a possible reason could be that excessive screen time and technology use in privileged families [51] contribute to visual impairments in children [52].

#### 4.2.4. Growth Status

Most Syrian children in Lebanon are living as refugees with limited access to basic needs, putting them at higher risk of malnutrition [53], which is a known environmental factor in a child’s physical development [54]. The association between low family income and parents’ low levels of education may reflect the same lack of nutritional security and healthy food practices.

Furthermore, only a significant correlation with having an ongoing treatment was found; we could speculate that certain drugs may interfere with the normal child’s development [55].

#### 4.2.5. Skin Inspection Findings

We showed an association between finding abusive signs in the children and the employment status of their fathers, disagreeing with other studies [56].

Skin diseases are a common problem in school children that can often be traced back to contact between classmates. The prevalence and pattern of these skin lesions tend to vary depending on the socioeconomic and cultural factors related to hygiene practices and attitudes towards seeking medical treatment [57]. Children with pre-existing medical conditions had lower rates of skin referrals because they are frequently monitored and exposed to healthcare professionals; therefore, skin lesions will be promptly addressed and managed.

#### 4.2.6. Posture Findings

Scoliosis is a common finding in the pediatric population, with an overall prevalence of 0.47–5.2% [58]. Our study revealed similar rates of abnormal posture (1.7%).

#### 4.2.7. Heart Auscultation Findings

Although most cardiac murmurs are benign, a murmur may be the only sign of serious heart disease in a pediatric population. This highlights the importance of cardiac auscultation screening for school-aged children. Comparable referral rates were shown in a large Chinese study [59]

#### 4.2.8. Lung Auscultation Findings

Wheezing, which could be asthma-related [60], accounts for the majority of abnormal lung auscultation referrals.

For instance, many young children experience wheezing during respiratory infections, especially with their increase in the winter season when our screening was conducted [61]. Therefore, referrals for further medical investigation are recommended. Furthermore, our data suggest an association between a higher maternal level of education with respiratory abnormalities, conflicting with multiple studies [62,63].

#### 4.2.9. Ear Exam Findings

Abnormal ear exam referrals were predominately filed for cerumen plugs (22% of students), while only limited cases are associated with serous otitis (1.7%) and hearing loss (1%). These ear problems were found to be more frequent among younger children, consistent with earlier studies [64]. Lower rates of cerumen impaction but higher rates of otitis were found in India [65]. A narrative review of global ear screening studies stated that there was a lack of true prevalence of hearing loss among school children, although referral percentages range from 0.16% in Taiwan to 15% in Brazil [66]. Our 1% rate of hearing loss falls in the better end of this range.

Furthermore, this referral was significantly associated with children from private schools. This is a reflection of socioeconomic status since children from wealthier backgrounds are more prone to damage their ears with easily accessible ear items such as ear buds and cotton tips, which have been linked to ear diseases [67,68].

#### 4.2.10. Pubertal Stage

In our study population, we did not investigate delayed puberty since the age group we considered is younger than 13 and 14 years [69]. However, students in our population exhibiting abnormal Tanner stage development were only assessed for possible precocious puberty. The latter is generally defined as the appearance of secondary sex characteristics before age 8 years in girls and before 9 years in boys [70]. This explains the predominant age group for this referral. In our study, boys were more referred for abnormal pubertal stage; however, this finding completely contradicts the well-known female-to-male ratio for precocious puberty (10:1) [70].

Our results suggest that a positive surgical history may impact the onset of puberty. This could be attributed to the hormonal changes that occur with surgery [71]. However, to our knowledge, no paper has yet been conducted to study the association between surgical history and precocious puberty.

#### 4.2.11. Neurological Exam Findings

Despite the limited number of referrals about this exam, our analysis revealed an association between going to a psychologist and having an abnormal neurologic exam. This finding agrees with the literature, which highlighted a higher risk of developing depression and anxiety problems in children with neurological disorders [72].

### 4.3. Mental Health Assessments

Nearly 2% of our students were marked for mental health referrals. A similar rate of children is identified for severe behavioral and emotional symptoms in a study using a universal screening system termed “The Behavior and Emotional Screening System” (BESS) [73]. It appears that our children were flagged for the mental health referral only when they displayed extreme signs requiring medical attention. However, milder signs also deserve to be addressed, seeing the importance of early intervention in mental health, according to the same article.

We also found that children with medical conditions were referred for mental health issues less often. These children probably receive greater attention from parents and healthcare professionals; therefore, their mental health needs are more likely to be recognized and addressed. This finding disagrees with previous research, which suggested that children with ongoing physical conditions are at higher risk for mental health issues [74].

### 4.4. Limitations

The strengths of the study reside in the large and diverse sample size and the range and variety of different health outcomes and behaviors investigated, from mental to physical health. In addition, the parent’s involvement helped us to identify certain implicated factors in the health of their children. However, there are several limitations to acknowledge. First, Baalbek-Hermel and South governorates were overrepresented compared to more populated governorates, limiting the generalizability of our findings. Additionally, we were limited by schools in the OML medical center territory, which can limit the representation of the entire population. Moreover, there is the potential bias concerning the referring doctor who is different in each school besides the social desirability bias that could affect parents’ and children’s interviews. However, the training of our doctors and nurses was conducted in the best way possible so their referrals would be as homogeneous as possible with one another! More importantly, our study cannot be subject to self-reporting bias since neither the student nor the parents had to fill out the questionnaire alone; eventually, there could be a misreporting on behalf of the parents (under-or over-reporting bias) who can mask (stigma, family income…) or exaggerate (in order to receive medications or medical assistance for their child health knowing the financial crisis affecting Lebanon). In addition, the misreporting bias on behalf of the children is possible (under or overreporting for mental assessment, since answers provided by both parties (parents and children) are neither anonymous nor totally confidential (medical doctors not only referred physical abnormalities but also mental ones). Overall, while this study can provide valuable insights into the health of school-aged children in Lebanon, it is important to interoperate the findings considering the limitations of our study.

### 4.5. Perspectives

All referrals were presently addressed to the OML centers; therefore, every child is currently benefiting from a medical follow-up to the nearest OML medical center! Additionally, all participating children were further registered in the OML patient database to benefit from the various medical services provided by health centers.

Our school health campaign purposely tackled and targeted very young children from 3 to 12 since the earlier healthy practices are implemented, the better the outcomes! Certainly, older age groups (13 to 18 years old) will also be studied, and other factors (drug, sexuality, alcohol, tobacco) will be assessed. This campaign will be repeated annually to verify that the procedures are running efficiently and leading to the desired objective. Furthermore, very critical socio-demographic characteristics were identified in this research. For instance, targeting the least educated family with low income, where both parents are working and the child is overweight, should be of high priority.

## 5. Conclusions

Our study demonstrated alarming results in terms of children’s medical conditions, mental health, and habits. Attention should be directed to dental care, weight problems, and mental health. It is also crucial to tackle routine vaccination compliance and address vision problems for better learning outcomes. This research recognizes the deficiencies existing in the school health policies and the imperative need for actionable measures to address the health situation in Lebanon, particularly in disadvantaged regions. Drawing inspiration from a study on Greek schools, a transformative policy plan is proposed, which advocates for, among other aspects, the integration of health services and inclusive education curricula, collaboration between the MoPH and the Ministry of Education, and the expansion of school nursing services [75]. These recommendations seek to bridge gaps and provide a fair and efficient school health system for the benefit of coming generations, with the aim of preventing the long-term contribution to adult morbidity and mortality.

## Figures and Tables

**Table 1:** Sociodemographic characteristics of participants.

			Frequency		Percentage	
**Nationality** Total = 7184	Lebanese		6682		93.0	
	Non-Lebanese		502		7.0	
		Syrian		480		6.7
		Palestinian		8		0.1
		Other		14		0.2
**Gender** Total = 7184	Male		3491		48.6	
	Female		3693		51.4	
**Age** Total = 7176	3–6 years		2654		37.0	
	7–9 years		2302		32.1	
	10–12 years		2220		30.9	
**Body Mass Index (BMI)** Total = 7184	Underweight		1926		26.8	
	Normal weight		2876		40.0	
	Overweight		2382		33.2	
**School type** Total = 7184	Public		2451		34.1	
	Private		4733		65.9	
**OML community health facilities** Total = 7184	Medical Mobile Units (MMUs)		1657		23.1	
	Medical centers		5527		76.9	
		Barqa		1002		13.9
		Khaldieh		608		8.5
		Zouk		1239		17.2
		Kobayat		524		7.3
		Room		122		1.7
		Ain El Remmaneh		294		4.1
		Siddikine		1374		19.1
		Kefraya		364		5.1
**Family Income** Total = 2992	No income		277		9.3	
	<100 USD		1228		41.0	
	100–300 USD		331		11.1	
	300–600 USD		902		30.1	
	600–900 USD		227		7.6	
	>900 USD		27		0.9	
**Mother’s occupation** Total = 3197	No work		3		0.1	
	Housewife		2249		70.3	
	Student		16		0.5	
	Unemployed		88		2.8	
	Employed		588		18.4	
	Self-employed		133		4.2	
	Retired		9		0.3	
	Disabled		2		0.1	
	Health field		76		2.4	
	Other		33		1.0	
**Father’s Occupation** Total = 3093	No work		8		0.3	
	Housewife		5		0.2	
	Student		11		0.4	
	Unemployed		213		6.9	
	Employed		1343		43.4	
	Self-employed		1110		35.9	
	Retired		118		3.8	
	Disabled		17		0.5	
	Health field		35		1.1	
	Other		233		7.5	
**Mother’s level of education** Total = 3238	No education		73		2.3	
	Primary		397		12.3	
	Complementary		722		22.3	
	Secondary		704		21.7	
	Undergraduate		436		13.5	
	University graduate		906		28.0	
**Father’s level of education** Total = 3115	No education		132		4.2	
	Primary		592		19.0	
	Complementary		953		30.6	
	Secondary		702		22.5	
	Undergraduate		283		9.1	
	University graduate		453		14.5	

**Table 2:** Participants’ medical history and medical status.

				Frequency		Percentage	
**Pregnancy and Birth**	Normal conditions Total = 3380	No		539		15.7	
		Yes		3844		84.1	
		Doesn’t know		7		0.2	
	Low birth weight Total = 3382	No		3101		91.7	
		Yes		262		7.7	
		Doesn’t know		19		0.6	
	Prematurity Total = 3382	No		3173		93.8	
		Yes		198		5.9	
		Doesn’t know		11		0.3	
	Childbirth difficulties Total = 3382	No		3092		91.4	
		Yes		274		8.1	
		Doesn’t know		16		0.5	
	Others Total = 3382	No		3220		95.2	
		Yes		153		4.5	
		Doesn’t know		9		0.3	
**Medical Conditions** **Currently or Previously Encountered**	Early puberty Total = 3380	No		3298		97.6	
		Yes		54		1.6	
		Doesn’t know		28		0.8	
	Diabetes Total = 3380	No		3350		99.1	
		Yes		11		0.3	
		Doesn’t know		19		0.6	
	Hypothyroidism Total = 3379	No		3328		98.5	
		Yes		21		0.6	
		Doesn’t know		30		0.9	
	Congenital heart disease Total = 3379	No		3338		98.8	
		Yes		29		0.9	
		Doesn’t know		12		0.4	
	Dyslipidemia Total = 3379	No		3343		98.9	
		Yes		10		0.3	
		Doesn’t know		26		0.8	
	Anemia Total = 3380	No		3158		93.4	
		Yes		146		4.3	
		Doesn’t know		76		2.2	
	Asthma Total = 3380	No		3161		93.5	
		Yes		196		5.8	
		Doesn’t know		23		0.7	
	Convulsions Total = 3380	No		3364		99.5	
		Yes		7		0.2	
		Doesn’t know		9		0.3	
	Developmental dysplasia of the hip Total = 3380	No		3363		99.5	
		Yes		7		0.2	
		Doesn’t know		10		0.3	
	Fractures Total = 3380	No		3166		93.7	
		Yes		209		6.2	
		Doesn’t know		5		0.1	
	Cancer Total = 3380	No		3368		99.6	
		Yes		8		0.2	
		Doesn’t know		4		0.1	
	Autoimmune disease(s) Total = 3379	No		3336		98.7	
		Yes		24		0.7	
		Doesn’t know		19		0.6	
	Genetic disorder(s) Total = 3380	No		3329		98.5	
		Yes		37		1.1	
		Doesn’t know		14		0.4	
	Chicken Pox (Varicella) Total = 3378	No		2770		82.0	
		Yes		528		15.6	
		Doesn’t know		80		2.4	
	German Measles (Rubella) Total = 3379	No		3192		94.5	
		Yes		105		3.1	
		Doesn’t know		82		2.4	
	Measles (Rubeola) Total = 3379	No		3188		94.3	
		Yes		112		3.3	
		Doesn’t know		79		2.3	
	Mumps Total = 3380	No		3195		94.5	
		Yes		125		3.7	
		Doesn’t know		60		1.8	
	Meningitis Total = 3377	No		3349		99.2	
		Yes		14		0.4	
		Doesn’t know		14		0.4	
	Recurrent ear infections Total = 3375	No		2893		85.7	
		Yes		453		13.4	
		Doesn’t know		29		0.9	
	Complicated urinary tract infections Total = 3377	No		3109		92.1	
		Yes		239		7.1	
		Doesn’t know		29		0.9	
	Any chronic or recurring pain Total = 3377	No		3241		96.0	
		Yes		123		3.6	
		Doesn’t know		13		0.4	
	Others Total = 3377	No		3069		90.9	
		Yes		301		8.9	
		Doesn’t know		7		0.2	
**Past Surgical History**	Hernia Total = 3379	No		3282		97.1	
		Yes		91		2.7	
		Doesn’t know		6		0.2	
	Surgery for undescended testis Total = 3378	No		3331		98.6	
		Yes		31		0.9	
		Doesn’t know		16		0.5	
	Correction of bone fractures Total = 3379	No		3269		96.7	
		Yes		106		3.1	
		Doesn’t know		4		0.1	
	Appendectomy Total = 3377	No		3364		99.6	
		Yes		10		0.3	
		Doesn’t know		3		0.1	
	Tonsillectomy Total = 3379	No		3312		98.0	
		Yes		64		1.9	
		Doesn’t know		3		0.1	
	Others Total = 3378	No		3211		95.1	
		Yes		165		4.9	
		Doesn’t know		2		0.1	
**Vaccination status** Total = 3290		Up-to-date		608		18.5	
		Not up-to-date		2619		79.6	
		Doesn’t Know		63		1.9	
**Ongoing Treatment** Total = 3331		No		2844		85.4	
		Yes		487		14.6	
**Support Device** (Prosthesis, hearing aid, etc.) Total = 3318		No		3245		97.8	
		Yes		73		2.2	
**Hospitalization**	Previous hospitalizations Total = 3326	No			1922		57.7
		Yes			1404		42.3
	Number of previous hospitalizations Total = 1407		1 time	799		56.7	
			2 times	278		19.7	
			3 times	134		9.5	
			4 times	53		3.8	
			5 times	47		3.3	
			6 times	41		2.9	
			7 times	55		3.9	
**Medical Examination**	Child visit to the pediatrician/doctor Total = 3297	No			680		20.6
		Yes			2617		79.4
	Number of child visits to the pediatrician/doctor per year Total = 2591		1 time	594		22.9	
			2 times	597		23.0	
			3 times	299		11.5	
			4 times	176		6.8	
			5 times	66		2.5	
			6 times	124		4.8	
			7 times	107		4.1	
			When needed	627		24.2	
	Previous child visit to the speech therapist Total = 3298	No		3128		94.8	
		Yes		170		5.2	
	Current child follow-up with the speech therapist Total = 3296	No		3257		98.8	
		Yes		39		1.2	
	Previous child visit to the psychologist Total = 3339	No		3196		95.7	
		Yes		143		4.3	
	Current child follow-up with psychologist Total = 3338	No		3303		99.0	
		Yes		35		1.0	
	Child visits to the dentist Total = 3338	Never		1627		48.7	
		Once per year		885		26.5	
		Twice per year		533		16.0	
		Once every 2 years		159		4.8	
		Once every 4 years		134		4.0	
**Hearing Situation**	Speaking loudly Total = 3362	No		2416		71.9	
		Yes		933		27.8	
		Doesn’t know		13		0.3	
	Repeat Total = 3362	No		2973		88.4	
		Yes		377		11.2	
		Doesn’t know		12		0.4	
	Turning up the TV volume Total = 3362	No		2827		84.1	
		Yes		531		15.8	
		Doesn’t know		4		0.1	
	Hearing well with noise around Total = 3357	No		500		14.9	
		Yes		2837		84.5	
		Doesn’t know		20		0.6	
	Understanding Total = 3355	No		272		8.1	
		Yes		3077		91.7	
		Doesn’t know		6		0.2	
	Detected hearing disorder Total = 3356	No		3252		96.9	
		Yes		104		3.1	
	Need for a hearing aid Total = 3356	No		659		98.4	
		Yes		5		0.7	
		Doesn’t know		6		0.9	
	Possession of one hearing aid Total = 360	No		354		98.3	
		Yes		6		1.7	
**Vision Situation**	Detected vision problem Total = 3353	No		2829		84.4	
		Yes		524		15.6	
	Myopia Total = 1117	No		827		74.0	
		Yes		221		19.8	
		Doesn’t know		69		6.2	
	Hyperopia Total = 1071	No		890		83.1	
		Yes		125		11.7	
		Doesn’t know		56		5.2	
	Strabismus Total = 1041	No		945		90.8	
		Yes		47		4.5	
		Doesn’t know		49		4.7	
	Eyes examination in the last 6 months Total = 3355	No		2668		79.5	
		Yes		687		20.5	
	Wearing eyeglasses Total = 3354	No		2992		89.2	
		Yes		362		10.8	
**Learning difficulties**	Presence of learning difficulties Total = 3352	No		2895		86.4	
		Yes		444		13.2	
		Doesn’t know		13		0.4	
	Dysphasia Total = 3352	No		3067		91.5	
		Yes		281		8.4	
		Doesn’t know		4		0.1	
	Dyslexia Total = 3353	No		3032		90.4	
		Yes		309		9.2	
		Doesn’t know		12		0.4	
	Dyspraxia Total = 3351	No		3268		97.5	
		Yes		75		2.2	
		Doesn’t know		8		0.2	
	Precocity Total = 3352	No		1722		51.4	
		Yes		1582		47.2	
		Doesn’t know		48		1.4	
	ADHD Total = 3351	No		3050		91.0	
		Yes		240		7.2	
		Doesn’t know		61		1.8	

**Table 3:** Participants’ habits and mental health.

Lifestyle Habits		Frequency		Percentage	
**Age of smoking initiation** Total = 7184	Never		6929		96.5
	<7 years		127		1.8
	8–9 years		57		0.8
	10–11 years		55		0.8
	12–13		16		0.2
**Number of days people smoked in the student’s presence during the past 7 days** Total = 7184	0		4372		60.9
	1–2		693		9.6
	3–4		540		7.5
	5–6		379		5.3
	7		1200		16.7
**Number of days the student used tobacco products during the past 30 days** Total = 7184	0		6597		91.8
	1–2		229		3.2
	3–5		111		1.5
	6–9		32		0.4
	10–19		28		0.4
	20–29		25		0.3
	30		162		2.3
**Number of days the student has had at least one alcoholic drink during the past 30 days** Total = 7184	0		6842		95.2
	1–2		233		3.2
	3–5		24		0.3
	6–9		35		0.5
	10–19		7		0.1
	20–29		6		0.1
	30		37		0.5
**Chance to try an illegal drug** (**even if the student did not try it**) Total = 7184	No		7128		99.2
	Yes		56		0.8
**Mental Health Assessment**		Frequency		Percentage	
**Homework checking by parents or guardians during the past 30 days** Total = 7184	Never		1799		25.0
	Rarely		218		3.0
	Sometimes		540		7.5
	Most of the time		939		13.1
	Always		3688		51.3
**Feeling that the parents or guardians understood their problems and worries during the past 30 days** Total = 7184	Never		2181		30.4
	Rarely		460		6.4
	Sometimes		655		9.1
	Most of the time		975		13.6
	Always		2913		40.5
**Feeling lonely during the past 12 months** Total = 7184	Never		5732		79.8
	Rarely		864		12.0
	Sometimes		407		5.7
	Most of the time		85		1.2
	Always		96		1.3
**Feeling so worried about something that they could not sleep at night during the past 12 months** Total = 7184	Never		5585		77.7
	Rarely		973		13.5
	Sometimes		474		6.6
	Most of the time		77		1.1
	Always		75		1.0
**Being sad or stressed during the past 12 months** Total = 7184	Never		5292		73.7
	Rarely		1084		15.1
	Sometimes		577		8.0
	Most of the time		184		2.6
	Always		47		0.7
**Being teased in a mean way or called hurtful names during the past 30 days** Total = 7184	Never		5470		76.1
	Rarely		879		12.2
	Sometimes		658		9.2
	Most of the time		125		1.7
	Always		52		0.7
**Number of days the student was bullied during the past 30 days** Total = 7184	0		5955		82.9
	1–2		679		9.5
	3–5		276		3.8
	6–9		103		1.4
	10–19		82		1.1
	20–29		34		0.5
	30		55		0.8
**Ways of bullying others most often performed by the student during the past 30 days, whether alone or as part of a group** Total = 7184	Did not bully		6420		89.4
	Hit, kick, push		319		4.4
	Made fun of their race		35		0.5
	Made fun of their religion		6		0.1
	Sexual		16		0.2
	Ignore		53		0.7
	Made fun of appearance		106		1.5
	Other ways		229		3.2

**Table 4:** Medical referrals to the OML medical centers based on abnormal examination findings.

Cause of Referral	Total	Frequency	Percentage
**Vital signs abnormalities**	7176	218	3
**Growth abnormalities**	7184	424	5.9
**Vision abnormalities**	7184	955	13.3
**Atypical lesions** (**signs of abuse**)	7184	43	0.6
**Infectious skin lesions** (**lice, scabies, mycosis, impetigo, varicella, measles, and other**)	7184	118	1.6
**Yellowish skin deposits**	7184	51	0.7
**Eye exam abnormal findings**	7184	114	1.6
**Postural abnormalities**	7184	120	1.7
**Heart auscultation abnormal findings**	7184	80	1.1
**Lung auscultation abnormal findings**	7184	83	1.2
**Thyroid exam abnormal findings**	7184	26	0.4
**Enlarged node or/and organ**	7184	50	0.7
**Otoscopy abnormal findings**	7184	312	4.3
**Neurological exam abnormal findings**	7184	45	0.6
**Oral exam abnormal findings**	7184	1259	17.5
**Mental health problem**	7184	120	1.7
**Incomplete vaccination**	6579	1670	25.4
**Early or late pubertal stage** **Girls: refer if <8y Stage II and higher** **Boys: refer if <9y stage II and higher**	- 7173	- 51	- 0.7
**Referred children with at least one of the above causes**	7184	3508	48.8

## Data Availability

The data presented in this study are available on request from the corresponding author. The data are not publicly available due to their containing information that could compromise the privacy of research participants.

## Supplementary Materials

The following supporting information can be downloaded at: https://www.mdpi.com/article/10.3390/children11020175/s1, Table S1: Associations between referrals & sociodemographic data and other factors; Table S2: Associations between referrals & sociodemographic data and other factors; Table S3: Associations between referrals & sociodemographic data and other factors.
