# Supplementary material for: Referrals and Determinant Factors of a National School Health Campaign in Lebanon on Children Aged between 3 and 12 Years Old
Source: Children (Basel). 2024 Jan 30;11(2):175. doi: 10.3390/children11020175 (PMC10886849; doi:10.3390/children11020175)
Supplement: Supplementary file 1 [file children-11-00175-s001.zip › children-2827129-supplementary.pdf]

children

MDPI

*Article*

# Referrals & Determinant Factors of a National School Health Campaign in Lebanon on Children Aged between 3 & 12 years-old

**Peter HABCHY et al.**

## Supplementary materials:

**Table S1A. Associations between referrals & sociodemographic data and other factors**

| Sociodemographic data \ Referrals | | Any referral N (%) | | Referral for abnormal growth N (%) | | Referral for abnormal vision N (%) | | Referral for mental health problem N (%) | | Referral for abnormal oral exam N (%) | | Referral for uncomplete Vaccination N (%) | |
|---|---|---|---|---|---|---|---|---|---|---|---|---|---|
| | | No | Yes | No | Yes | No | Yes | No | Yes | No | Yes | No | Yes |
| Nationality | Lebanese | 3357 (50.2) | 3325 (49.8) | 6303 (94.3) | 379 (5.7) | 5762 (86.2) | 920 (13.8) | 6569 (98.3) | 113 (1.7) | 5482 (82.0) | 1200 (18.0) | 4522 (74.3) | 1561 (25.7) |
| | Syrian | 309 (64.4) | 171 (35.6) | 437 (91.0) | 43 (9.0) | 449 (93.5) | 31 (6.5) | 474 (98.8) | 6 (1.3) | 425 (88.5) | 55 (11.5) | 373 (78.5) | 102 (21.5) |
| | Palestinian | 6 (75.0) | 2 (25) | 8 (100.0) | 0 (0.0) | 7 (87.5) | 1 (12.5) | 8 (100.0) | 0 (0.0) | 8 (100.0) | 0 (0.0) | 6 (75.0) | 2 (25.0) |
| | Other | 4 (28.6) | 10 (71.4) | 12 (85.7) | 2 (14.3) | 11 (78.6) | 3 (21.4) | 13 (92.9) | 1 (7.1) | 10 (71.4) | 4 (28.6) | 8 (61.5) | 5 (38.5) |
| | | | p* | | p* | | p* | | NS | | p* | | NS |
| Gender | Male | 1830 (52.4) | 1661 (47.6) | 3289 (94.2) | 202 (5.8) | 3059 (87.6) | 432 (12.4) | 3429 (98.2) | 62 (1.8) | 2890 (82.8) | 601 (17.2) | 2390 (74.7) | 809 (25.3) |
| | Female | 1846 (50.0) | 1847 (50.0) | 3471 (94.0) | 222 (6.0) | 3170 (85.8) | 523 (14.2) | 3635 (98.4) | 58 (1.6) | 3035 (82.2) | 658 (17.8) | 2519 (74.5) | 861 (25.5) |
| | | | p* | | NS | | p* | | NS | | NS | | NS |
| Age | 3-6 years | 1589 (59.8) | 1068 (40.2) | 2561 (96.4) | 96 (3.6) | 2515 (94.7) | 142 (5.3) | 2622 (98.7) | 35 (1.3) | 2282 (85.9) | 375 (14.1) | 1899 (79.8) | 482 (20.2) |
| | 7-9 years | 1104 (47.9) | 1201 (52.1) | 2136 (92.7) | 169 (7.3) | 1924 (83.5) | 381 (16.5) | 2271 (98.5) | 34 (1.5) | 1851 (80.3) | 454 (19.7) | 1624 (74.1) | 569 (25.9) |
| | 10-12 years | 983 (44.2) | 1239 (55.8) | 2063 (92.8) | 159 (7.2) | 1790 (80.6) | 432 (19.4) | 2171 (97.7) | 51 (2.3) | 1792 (80.6) | 430 (19.4) | 1386 (69.1) | 619 (30.9) |
| | | | p* | | p* | | p* | | p* | | p* | | p* |
| BMI | Underweight | 1064 (55.2) | 862 (44.8) | 1794 (93.1) | 132 (6.9) | 1687 (87.6) | 239 (12.4) | 1892 (98.2) | 34 (1.8) | 1604 (83.3) | 322 (16.7) | 1369 (77.7) | 392 (22.3) |
| | Normal weight | 1522 (52.9) | 1354 (47.1) | 2745 (95.4) | 131 (4.6) | 2552 (88.7) | 324 (11.3) | 2838 (98.7) | 38 (1.3) | 2387 (83.0) | 489 (17.0) | 1960 (74.6) | 669 (25.4) |
| | Overweight | 1090 (45.8) | 1292 (54.2) | 2221 (93.2) | 161 (6.8) | 1990 (83.5) | 392 (16.5) | 2334 (98.0) | 48 (2.0) | 1934 (81.2) | 44 (18.8) | 1580 (72.2) | 609 (27.8) |
| | | | p* | | p* | | p* | | NS | | NS | | p* |
| Governorate | Beirut | 93 (31.6) | 201 (68.4) | 269 (91.5) | 25 (8.5) | 245 (83.3) | 49 (16.7) | 293 (99.7) | 1 (0.3) | 222 (75.5) | 72 (24.5) | 187 (64.0) | 105 (36.0) |
| | Mount Lebanon | 653 (48.6) | 692 (51.4) | 153 (90.0) | 17 (10.0) | 152 (89.4) | 18 (10.6) | 148 (87.1) | 22 (12.9) | 127 (74.7) | 43 (25.3) | 101 (61.2) | 64 (38.8) |
| | North/Akkar | 673 (55.9) | 532 (44.1) | 1125 (93.4) | 80 (6.6) | 1103 (91.5) | 102 (8.5) | 1196 (99.3) | 9 (0.7) | 1093 (90.7) | 112 (9.3) | 760 (71.4) | 305 (28.6) |
| | Beqaa/Baalbek | 1855 (54.8) | 1527 (45.2) | 3201 (94.6) | 181 (5.4) | 2869 (84.8) | 513 (15.2) | 3334 (98.6) | 48 (1.4) | 2710 (80.1) | 672 (19.9) | 2405 (81.5) | 547 (18.5) |
| | South | 976 (45.8) | 1157 (54.2) | 2012 (94.3) | 121 (5.7) | 1860 (87.2) | 273 (12.8) | 2093 (98.1) | 40 (1.9) | 1773 (83.1) | 360 (16.9) | 1456 (69.2) | 649 (30.8) |
| | | | p* | | p* | | p* | | p* | | p* | | p* |
| School type | Public | 1194 (48.7) | 1257 (51.3) | 2264 (92.4) | 187 (7.6) | 2181 (89.0) | 270 (11.0) | 2407 (98.2) | 44 (1.8) | 2029 (82.8) | 422 (17.2) | 1543 (63.9) | 873 (36.1) |
| | Private | 2482 (52.4) | 2251 (47.6) | 4496 (95.0) | 237 (5.0) | 4048 (85.5) | 685 (14.5) | 4657 (98.4) | 76 (1.6) | 3896 (82.3) | 837 (17.7) | 3366 (80.9) | 797 (19.1) |
| | | | p* | | p* | | p* | | NS | | NS | | p* |
| Family income | No income | 138 (49.8) | 139 (50.2) | 261 (94.2) | 16 (5.8) | 235 (84.8) | 42 (15.2) | 275 (99.3) | 2 (0.7) | 231 (83.4) | 46 (16.6) | 184 (70.5) | 77 (29.5) |
| | <100$ | 567 (46.1) | 664 (53.9) | 1128 (91.6) | 103 (8.4) | 1071 (87.0) | 160 (13.0) | 1212 (98.5) | 19 (1.5) | 958 (77.8) | 273 (22.2) | 754 (67.6) | 361 (32.4) |
| | 100-300$ | 165 (49.7) | 167 (50.3) | 312 (94.0) | 20 (6.0) | 288 (86.7) | 44 (13.3) | 325 (97.9) | 7 (2.1) | 263 (79.2) | 69 (20.8) | 215 (72.1) | 83 (27.9) |
| | 300-600$ | 422 (46.8) | 480 (53.2) | 859 (95.2) | 43 (4.8) | 741 (82.2) | 161 (17.8) | 889 (98.6) | 13 (1.4) | 730 (80.9) | 172 (19.1) | 613 (73.1) | 226 (26.9) |
| | 600-900$ | 100 (44.1) | 127 (55.9) | 223 (98.2) | 4 (1.8) | 181 (79.7) | 46 (20.3) | 221 (97.4) | 6 (2.6) | 193 (85.0) | 34 (15.0) | 162 (80.6) | 39 (19.4) |
| | >900$ | 12 (44.4) | 15 (55.6) | 26 (96.3) | 1 (3.7) | 22 (81.5) | 5 (18.5) | 27 (100.0) | 0 (0.0) | 25 (92.6) | 2 (7.4) | 19 (73.1) | 7 (26.9) |
| | | | NS | | p* | | p* | | NS | | p* | | p* |
| Mother's occupation | No work | 3 (100.0) | 0 (0.0) | 3 (100.0) | 0 (0.0) | 3 (100.0) | 0 (0.0) | 3 (100.0) | 0 (0.0) | 3 (100.0) | 0 (0.0) | 2 (100.0) | 0 (0.0) |
| | Housewife | 1114 (49.5) | 1137 (50.5) | 2103 (93.4) | 148 (6.6) | 1935 (86.0) | 316 (14.0) | 2216 (98.4) | 35 (1.6) | 1819 (80.8) | 432 (19.2) | 1487 (71.8) | 583 (28.2) |
| | Student | 12 (75.0) | 4 (25.0) | 16 (100.0) | 0 (0.0) | 13 (81.3) | 3 (18.8) | 16 (100.0) | 0 (0.0) | 15 (93.8) | 1 (6.3) | 10 (76.9) | 3 (23.1) |
| | Employed | 292 (49.6) | 297 (50.4) | 567 (96.3) | 22 (3.7) | 491 (83.4) | 98 (16.6) | 574 (97.5) | 15 (2.5) | 494 (83.9) | 95 (16.1) | 437 (81.8) | 97 (18.2) |
| | Self-employed | 68 (50.7) | 66 (49.3) | 125 (93.3) | 9 (6.7) | 117 (87.3) | 17 (12.7) | 133 (99.3) | 1 (0.7) | 111 (82.8) | 23 (17.2) | 85 (69.7) | 37 (30.3) |
| | Unemployed | 44 (50.0) | 44 (50.0) | 86 (97.7) | 2 (2.3) | 77 (87.5) | 11 (12.5) | 87 (98.9) | 1 (1.1) | 77 (87.5) | 11 (12.5) | 58 (69.0) | 26 (31.0) |
| | Retired | 9 (100.0) | 0 (0.0) | 9 (100.0) | 0 (0.0) | 9 (100.0) | 0 (0.0) | 9 (100.0) | 0 (0.0) | 9 (100.0) | 0 (0.0) | 9 (100.0) | 0 (0.0) |
| | Disabled | 1 (50.0) | 1 (50.0) | 2 (100.0) | 0 (0.0) | 1 (50.0) | 1 (50.0) | 2 (100.0) | 0 (0.0) | 2 (100.0) | 0 (0.0) | 2 (100.0) | 0 (0.0) |
| | Health field | 47 (61.8) | 29 (38.2) | 75 (98.7) | 1 (1.3) | 70 (92.1) | 6 (7.9) | 76 (100.0) | 0 (0.0) | 69 (90.8) | 7 (9.2) | 56 (81.2) | 13 (18.8) |
| | Other | 14 (42.4) | 19 (57.6) | 33 (100.0) | 0 (0.0) | 29 (87.9) | 4 (12.1) | 31 (93.9) | 2 (6.1) | 26 (78.8) | 7 (21.2) | 21 (72.4) | 8 (27.6) |
| | | | p* | | NS | | NS | | NS | | NS | | p* |
| Father's occupation | No work | 7 (87.5) | 1 (12.5) | 8 (100.0) | 0 (0.0) | 8 (100.0) | 0 (0.0) | 8 (100.0) | 0 (0.0) | 8 (100.0) | 0 (0.0) | 7 (87.5) | 1 (12.5) |
| | Housewife | 3 (60.0 | 2 (40.0) | 5 (100.0) | 0 (0.0) | 5 (100.0) | 0 (0.0) | 5 (100.0) | 0 (0.0) | 4 (80.0) | 1 (20.0) | 3 (60.0) | 2 (40.0) |
| | Student | 9 (81.8) | 2 (18.2) | 11 (100.0) | 0 (0.0) | 11 (100.0) | 0 (0.0) | 11 (100.0) | 0 (0.0) | 11 (100.0) | 0 (0.0) | 8 (80.0 | 2 (20.0) |
| | Employed | 693 (51.5) | 652 (48.5) | 1277 (94.9) | 68 (5.1) | 1157 (86.0) | 188 (14.0) | 1321 (98.2) | 24 (1.8) | 1102 (81.9) | 243 (18.1) | 940 (78.0) | 265 (22.0) |

| | | | | | | | | | | | | | |
|---|---|---|---|---|---|---|---|---|---|---|---|---|---|
| | **Self-employed** | 507 (45.7) | 603 (54.3) | 1036 (93.3) | 74 (6.7) | 946 (85.2) | 164 (14.8) | 1091 (98.3) | 19 (1.7) | 910 (82.0) | 200 (18.0) | 708 (67.9) | 335 (32.1) |
| | **Unemployed** | 117 (54.9) | 96 (45.1) | 203 (95.3) | 10 (4.7) | 183 (85.9) | 30 (14.1) | 211 (99.1) | 2 (0.9) | 185 (86.9) | 28 (13.1) | 145 (72.1) | 56 (27.9) |
| | **Retired** | 58 (49.2) | 60 (50.8) | 111 (94.1) | 7 (5.9) | 97 (82.2) | 21 (17.8) | 115 (97.5) | 3 (2.5) | 90 (76.3) | 28 (23.7) | 79 (79.0) | 21 (21.0) |
| | **Disabled** | 11 (64.7) | 6 (35.3) | 16 (94.1) | 1 (5.9) | 16 (94.1) | 1 (5.9) | 17 (100.0) | 0 (0.0) | 14 (82.4) | 3 (17.6) | 13 (76.5) | 4 (23.5) |
| | **Health field** | 23 (65.7) | 12 (34.3) | 33 (94.3) | 2 (5.7) | 32 (91.4) | 3 (8.6) | 35 (100.0) | 0 (0.0) | 32 (91.4) | 3 (8.6) | 30 (88.2) | 4 (11.8) |
| | **Other** | 122 (52.1) | 112 (47.9) | 219 (93.6) | 15 (6.4) | 202 (86.3) | 32 (13.7) | 231 (98.7) | 3 (1.3) | 179 (76.5) | 55 (23.5) | 157 (71.4) | 63 (28.6) |
| | | | **p*** | | **NS** | | **NS** | | **NS** | | **p*** | | **p*** |
| **Mother's level of education** | **No education** | 30 (41.1) | 43 (58.9) | 67 (91.8) | 6 (8.2) | 61 (83.6) | 12 (16.4) | 73 (100.0) | 0 (0.0) | 53 (72.6) | 20 (27.4) | 42 (60.9) | 27 (39.1) |
| | **Primary** | 184 (46.1) | 215 (53.9) | 362 (90.7) | 37 (9.3) | 351 (88.0) | 48 (12.0) | 392 (98.2) | 7 (1.8) | 312 (78.2) | 87 (21.8) | 249 (64.8) | 135 (35.2) |
| | **Complementary** | 346 (47.9) | 376 (52.1) | 674 (93.4) | 48 (6.6) | 626 (86.7) | 96 (13.3) | 707 (97.9 | 15 (2.1) | 588 (81.4) | 134 (18.6) | 456 (69.2) | 203 (30.8) |
| | **Secondary** | 336 (47.7) | 369 (52.3) | 661 (93.8) | 44 (6.2) | 574 (81.4) | 131 (18.6) | 691 (98.0) | 14 (2.0) | 565 (80.1) | 140 (19.9) | 472 (73.4) | 171 (26.6) |
| | **Undergraduate** | 235 (53.9) | 201 (46.1) | 421 (96.6) | 15 (3.4) | 376 (86.2) | 60 (13.8) | 432 (99.1) | 4 (0.9) | 362 (83.0) | 74 (17.0) | 321 (81.5) | 73 (18.5) |
| | **University graduate** | 497 (54.8) | 410 (45.2) | 873 (96.3) | 34 (3.7) | 793 (87.4) | 114 (12.6) | 893 (98.5) | 14 (1.5) | 773 (85.2) | 134 (14.8) | 654 (79.2) | 172 (20.8) |
| | | | **p*** | | **p*** | | **p*** | | **NS** | | **p*** | | **p*** |
| **Father's level of education** | **No education** | 77 (58.3) | 55 (41.7) | 124 (93.9) | 8 (6.1) | 121 (91.7) | 11 (8.3) | 130 (98.5) | 2 (1.5) | 116 (87.9) | 16 (12.1) | 84 (68.9) | 38 (31.1) |
| | **Primary** | 283 (47.8) | 309 (52.2) | 538 (90.9) | 54 (9.1) | 519 (87.7) | 73 (12.3) | 582 (98.3) | 10 (1.7) | 474 (80.1) | 118 (19.9) | 375 (67.9) | 177 (32.1) |
| | **Complementary** | 435 (45.5) | 520 (54.5) | 882 (92.4) | 73 (7.6) | 810 (84.8) | 145 (15.2) | 936 (98.0) | 19 (2.0) | 759 (79.5) | 196 (20.5) | 606 (69.0) | 272 (31.0) |
| | **Secondary** | 351 (49.9) | 352 (50.1) | 676 (96.2) | 27 (3.8) | 576 (81.9) | 127 (18.1) | 690 (98.2) | 13 (1.8) | 580 (82.5) | 123 (17.5) | 490 (78.1) | 137 (21.9) |
| | **Undergraduate** | 152 (53.7) | 131 (46.3) | 273 (96.5) | 10 (3.5) | 249 (88.0) | 34 (12.0) | 280 (98.9) | 3 (1.1) | 227 (80.2) | 56 (19.8) | 207 (80.2) | 51 (19.8) |
| | **University graduate** | 261 (57.6) | 192 (42.4) | 445 (98.2) | 8 (1.8) | 399 (88.1) | 54 (11.9) | 449 (99.1) | 4 (0.9) | 395 (87.2) | 58 (12.8) | 339 (79.4) | 88 (20.6) |
| | | | **p*** | | **p*** | | **p*** | | **NS** | | **p*** | | **p*** |
| **Other factors** | | | | | | | | | | | | | |
| **Medical history** | **No** | 838 (49.4) | 858 (50.6) | 1603 (94.5) | 93 (5.5) | 1449 (85.4) | 247 (14.6) | 1658 (97.8) | 38 (2.2) | 1364 (80.4) | 332 (19.6) | 1155 (74.7) | 392 (25.3) |
| | **Yes** | 831 (49.3) | 853 (50.7) | 1568 (93.1) | 116 (6.9) | 1452 (86.2) | 232 (13.8) | 1664 (98.8) | 20 (1.2) | 1385 (82.2) | 299 (17.8) | 1096 (70.9) | 449 (29.1) |
| | | | **NS** | | **NS** | | **NS** | | **p*** | | **NS** | | **p*** |
| **Ongoing treatment** | **No** | 1400 (49.2) | 1444 (50.8) | 2661 (93.6) | 183 (6.4) | 2434 (85.6) | 410 (14.4) | 2790 (98.1) | 54 (1.9) | 2334 (82.1) | 510 (17.9) | 1888 (72.5) | 717 (27.5) |
| | **Yes** | 240 (49.3) | 247 (50.7) | 469 (96.3) | 18 (3.7) | 430 (88.3) | 57 (11.7) | 480 (98.6) | 7 (1.4) | 384 (78.9) | 103 (21.1) | 341 (76.5) | 105 (23.5) |
| | | | **NS** | | **p*** | | **NS** | | **NS** | | **NS** | | **NS** |
| **Child visit to the pediatrician/doctor** | **No** | 291 (42.9) | 388 (57.1) | 632 (93.1) | 47 (6.9) | 562 (82.8) | 117 (17.2) | 664 (97.8) | 15 (2.2) | 548 (80.7) | 131 (19.3) | 435 (70.4) | 183 (29.6) |
| | **Yes** | 1330 (50.8) | 1288 (49.2) | 2466 (94.2) | 152 (5.8) | 2270 (86.7) | 348 (13.3) | 2572 (98.2) | 46 (1.8) | 2139 (81.7) | 479 (18.3) | 1772 (73.7) | 631 (26.3) |
| | | | **p*** | | **NS** | | **p*** | | **NS** | | **NS** | | **NS** |
| **Previous child visit to the psychologist** | **No** | 1608 (50.3) | 1588 (49.7) | 3008 (94.1) | 188 (5.9) | 2751 (86.1) | 445 (13.9) | 3147 (98.5) | 49 (1.5) | 2618 (81.9) | 578 (18.1) | 2163 (73.6) | 777 (26.4) |
| | **Yes** | 62 (43.4) | 81 (56.6) | 135 (94.4) | 8 (5.6) | 113 (79.0) | 30 (21.0) | 138 (96.5) | 5 (3.5) | 115 (80.4) | 28 (19.6) | 89 (68.5) | 41 (31.5) |
| | | | **NS** | | **NS** | | **p*** | | **NS** | | **NS** | | **NS** |

**p* = p < 0.05**
**NS = Non-significant p value > 0.05**

## Table S1B. Associations between referrals & sociodemographic data and other factors

| Referrals / Sociodemographic factors | | **Referral for abnormal vital signs N (%)** | | **Referral for atypical skin lesions (signs of abuse) N (%)** | | **Referral for infectious skin lesions N (%)** | | **Referral for abnormal posture N (%)** | | **Referral for abnormal heart auscultation N (%)** | |
|---|---|---|---|---|---|---|---|---|---|---|---|
| | | No | Yes | No | Yes | No | Yes | No | Yes | No | Yes |
| **Nationality** | **Lebanese** | 6469 (96.9) | 207 (3.1) | 6645 (99.4) | 37 (0.6) | 6572 (98.4) | 110 (1.6) | 6567 (98.3) | 115 (1.7) | 6606 (98.9) | 76 (1.1) |
| | **Syrian** | 468 (97.9) | 10 (2.1) | 474 (98.8) | 6 (1.3) | 474 (98.8) | 6 (1.3) | 475 (99.0) | 5 (1.0) | 476 (99.2) | 4 (0.8) |
| | **Palestinian** | 8 (100.0) | 0 (0.0) | 8 (100.0) | 0 (0.0) | 8 (100.0) | 0 (0.0) | 8 (100.0) | 0 (0.0) | 8 (100.0) | 0 (0.0) |
| | **Other** | 13 (92.9) | 1 (7.1) **NS** | 14 (100.0) | 0 (0.0) **NS** | 12 (85.7) | 2 (14.3) **p*** | 14 (100.0) | 0 (0.0) **NS** | 14 (100.0) | 0 (0.0) **NS** |
| **Gender** | **Male** | 3391 (97.2) | 98 (2.8) | 3467 (99.3) | 24 (0.7) | 3448 (98.8) | 43 (1.2) | 3450 (98.8) | 41 (1.2) | 3453 (98.9) | 38 (1.1) |
| | **Female** | 3567 (96.7) | 120 (3.3)**NS** | 3674 (99.5) | 19 (0.5) **NS** | 3618 (98.0) | 75 (2.0) **p*** | 3614 (97.9) | 79 (2.1) **p*** | 3651 (98.9) | 42 (1.1) **NS** |
| **Age** | **3-6 years** | 2588 (97.5) | 66 (2.5) | 2641 (99.4) | 16 (0.6) | 2610 (98.2) | 47 (1.8) | 2635 (99.2) | 22 (0.8) | 2622 (98.7) | 35 (1.3) |
| | **7-9 years** | 2238 (97.2) | 64 (2.8) | 2288 (99.3) | 17 (0.7) | 2259 (98.0) | 46 (2.0) | 2257 (97.9) | 48 (2.1) | 2280 (98.9) | 25 (1.1) |
| | **10-12 years** | 2132 (96.0) | 88 (4.0) **p*** | 2212 (99.5) | 10 (0.5) **NS** | 2197 (98.9) | 25 (1.1) **NS** | 2172 (97.7) | 50 (2.3) **p*** | 2202 (99.1) | 20 (0.9) **NS** |
| **BMI** | **Underweight** | 1895 (98.4) | 31 (1.6) | 1916 (99.5) | 10 (0.5) | 1904 (98.9) | 22 (1.1) | 1907 (99.0) | 19 (1.0) | 1904 (98.9) | 22 (1.1) |
| | **Normal weight** | 2784 (96.9) | 88 (3.1) | 2864 (99.6) | 12 (0.4) | 2828 (98.3) | 48 (1.7) | 2825 (98.2) | 51 (1.8) | 2844 (98.9) | 32 (1.1) |
| | **Overweight** | 2542 (95.8) | 99 (4.2) **p*** | 2361 (99.1) | 21 (1.9) **NS** | 2334 (98.0) | 48 (2.0) **NS** | 2332 (97.9) | 50 (2.1) **p*** | 2356 (98.9) | 26 (1.1) **NS** |
| **Governorate** | **Beirut** | 276 (93.9) | 18 (6.1) | 291 (99.0) | 3 (1.0) | 278 (94.6) | 16 (5.4) | 292 (99.3) | 2 (0.7) | 288 (98.0) | 6 (2.0) |
| | **Mount Lebanon** | 159 (93.5) | 11(6.5) | 167 (98.2) | 3 (1.8) | 162 (95.3) | 8 (4.7) | 160 (94.1) | 10 (5.9) | 168 (98.8) | 2 (1.2) |
| | **North/Akkar** | 1162 (96.7) | 40 (3.3) | 1193 (99.0) | 12 (1.0) | 1180 (97.9) | 25 (2.1) | 1189 (98.7) | 16 (1.3) | 1183 (98.2) | 22 (1.8) |
| | **Beqaa/Baalbek** | 3281 (97.1) | 98 (2.9) | 3367 (99.6) | 15 (0.4) | 3327 (98.4) | 55 (1.6) | 3332 (98.5) | 50 (1.5) | 3356 (99.2) | 26 (0.8) |
| | **South** | 2080 (97.6) | 51 (2.4) **p*** | 2123 (99.5) | 10 (0.5) **p*** | 2119 (99.3) | 14 (0.7) **p*** | 2091 (98.0) | 42 (2.0) **p*** | 2109 (98.9) | 24 (1.1) **p*** |
| **School type** | **Public** | 2334 (95.3) | 114 (4.7) | 2430 (99.1) | 21 (0.9) | 2395 (97.7) | 56 (2.3) | 2421 (98.8) | 30 (1.2) | 2419 (98.7) | 32 (1.3) |
| | **Private** | 4624 (97.8) | 104 (2.2) **p*** | 4711 (99.5) | 22 (0.5) **p*** | 4671 (98.7) | 62 (1.3) **p*** | 4643 (98.1) | 90 (1.9) **p*** | 4685 (99.0) | 48 (1.0) **NS** |
| **Family income** | **No income** | 267 (96.4) | 10 (3.6) | 276 (99.6) | 1 (0.4) | 273 (98.6) | 4 (1.4) | 270 (97.5) | 7 (2.5) | 274 (98.9) | 3 (1.1) |
| | **<100$** | 1169 (95.2) | 59 (4.8) | 1223 (99.4) | 8 (0.6) | 1205 (97.9) | 26 (2.1) | 1211 (98.4) | 20 (1.6) | 1217 (98.9) | 14 (1.1) |
| | **100-300$** | 324 (97.9) | 7 (2.1) | 329 (99.1) | 3 (0.9) | 323 (97.3) | 9 (2.7) | 325 (97.9) | 7 (2.1) | 329 (99.1) | 3 (0.9) |
| | **300-600$** | 881 (97.7) | 21 (2.3) | 898 (99.6) | 4 (0.4) | 887 (98.3) | 15 (1.7) | 883 (97.9) | 19 (2.1) | 891 (98.8) | 11 (1.2) |
| | **600-900$** | 222 (97.8) | 5 (2.2) | 225 (99.1) | 2 (0.9) | 222 (97.8) | 5 (2.2) | 225 (99.1) | 2 (0.9) | 226 (99.6) | 1 (0.4) |
| | **>900$** | 26 (96.3) | 1 (3.7) **p*** | 26 (96.3) | 1 (3.7) **NS** | 27 (100.0) | 0 (0.0) **NS** | 25 (92.6) | 2 (7.4) **NS** | 27 (100.0) | 0 (0.0) **NS** |
| **Mother's occupation** | **No work** | 3 (100.0) | 0 (0.0) | 3 (100.0) | 0 (0.0) | 3 (100.0) | 0 (0.0) | 3 (100.0) | 0 (0.0) | 3 (100.0) | 0 (0.0) |
| | **Housewife** | 2168 (96.4) | 81 (3.6) | 2237 (99.4) | 14 (0.6) | 2219 (98.6) | 32 (1.4) | 2208 (98.1) | 43 (1.9) | 2230 (99.1) | 21 (0.9) |

| | | | | | | | | | | | |
|---|---|---|---|---|---|---|---|---|---|---|---|
| | **Student** | 16 (100.0) | 0 (0.0) | 16 (100.0) | 0 (0.0) | 16 (100.0) | 0 (0.0) | 15 (93.8) | 1 (6.3) | 16 (100.0) | 0 (0.0) |
| | **Employed** | 571 (97.1) | 17 (2.9) | 584 (99.2) | 5 (0.8) | 574 (97.5) | 15 (2.5) | 580 (98.5) | 9 (1.5) | 582 (98.8) | 7 (1.2) |
| | **Self-employed** | 130 (97.7) | 3 (2.3) | 134 (100.0) | 0 (0.0) | 132 (98.5) | 2 (1.5) | 132 (98.5) | 2 (1.5) | 131 (97.8) | 3 (2.2) |
| | **Unemployed** | 88 (100.0) | 0 (0.0) | 87 (98.9) | 1 (1.1) | 86 (97.7) | 2 (2.3) | 88 (100.0) | 0 (0.0) | 88 (100.0) | 0 (0.0) |
| | **Retired** | 9 (100.0) | 0 (0.0) | 9 (100.0) | 0 (0.0) | 9 (100.0) | 0 (0.0) | 9 (100.0) | 0 (0.0) | 9 (100.0) | 0 (0.0) |
| | **Disabled** | 2 (100.0) | 0 (0.0) | 2 (100.0) | 0 (0.0) | 2 (100.0) | 0 (0.0) | 2 (100.0) | 0 (0.0) | 2 (100.0) | 0 (0.0) |
| | **Health field** | 76 (100.0 | 0 (0.0) | 76 (100.0) | 0 (0.0) | 74 (97.4) | 2 (2.6) | 74 (97.4) | 2 (2.6) | 75 (98.7) | 1 (1.3) |
| | **Other** | 31 (93.9) | 2 (6.1) **NS** | 33 (100.0) | 0 (0.0) **NS** | 30 (90.9) | 3 (9.1) **NS** | 33 (100.0) | 0 (0.0) **p*** | 32 (97.0) | 1 (3.0) **NS** |
| **Father's occupation** | **No work** | 8 (100.0) | 0 (0.0) | 8 (100.0) | 0 (0.0) | 8 (100.0) | 0 (0.0) | 8 (100.0) | 0 (0.0) | 7 (87.5) | 1 (12.5) |
| | **Housewife** | 5 (100.0) | 0 (0.0) | 5 (100.0) | 0 (0.0) | 5 (100.0) | 0 (0.0) | 5 (100.0) | 0 (0.0) | 5 (100.0) | 0 (0.0) |
| | **Student** | 11 (100.0) | 0 (0.0) | 11 (100.0) | 0 (0.0) | 11 (100.0) | 0 (0.0) | 11 (100.0) | 0 (0.0) | 11 (100.0) | 0 (0.0) |
| | **Employed** | 1311 (97.6) | 32 (2.4) | 1338 (99.5) | 7 (0.5) | 1325 (98.5) | 20 (1.5) | 1328 (98.7) | 17 (1.3) | 1333 (99.1) | 12 (0.9) |
| | **Self-employed** | 1075 (96.8) | 35 (3.2) | 1103 (99.4) | 7 (0.6) | 1091 (98.3) | 19 (1.7) | 1083 (97.6) | 27 (2.4) | 1100 (99.1) | 10 (0.9) |
| | **Unemployed** | 205 (96.2) | 8 (3.8) | 212 (99.5) | 1 (0.5) | 211 (99.1) | 2 (0.9) | 210 (98.6) | 3 (1.4) | 209 (98.1) | 4 (1.9) |
| | **Retired** | 106 (89.8) | 12 (10.2) | 115 (97.5) | 3 (2.5) | 117 (99.2) | 1 (0.8) | 115 (97.5) | 3 (2.5) | 118 (100.0) | 0 (0.0) |
| | **Disabled** | 17 (100.0) | 0 (0.0) | 16 (94.1) | 1 (5.9) | 17 (100.0) | 0 (0.0) | 17 (100.0) | 0 (0.0) | 17 (100.0) | 0 (0.0) |
| | **Health field** | 35 (100.0) | 0 (0.0) | 35 (100.0) | 0 (0.0) | 33 (94.3) | 2 (5.7) | 35 (100.0) | 0 (0.0) | 34 (97.1) | 1 (2.9) |
| | **Other** | 221 (94.8) | 12 (5.2) **p*** | 234 (100.0) | 0 (0.0) **p*** | 228 (97.4) | 6 (2.6) **NS** | 230 (98.3) | 4 (1.7) **NS** | 231 (98.7) | 3 (1.3) **NS** |
| **Mother's level of education** | **No education** | 70 (95.9) | 3 (4.1) | 73 (100.0) | 0 (0.0) | 71 (97.3) | 2 (2.7) | 73 (100.0) | 0 (0.0) | 73 (100.0) | 0 (0.0) |
| | **Primary** | 381 (96.0) | 16 (4.0) | 394 (98.7) | 5 (1.3) | 391 (98.0) | 8 (2.0 | 393 (98.5) | 6 (1.5) | 394 (98.7) | 5 (1.3) |
| | **Complementary** | 699 (96.8) | 23 (3.2) | 718 (99.4) | 4 (0.6) | 704 (97.5) | 18 (2.5) | 707 (97.9) | 15 (2.1) | 715 (99.0) | 7 (1.0) |
| | **Secondary** | 680 (96.6) | 24 (3.4) | 702 (99.6) | 3 (0.4) | 696 (98.7) | 9 (1.3) | 693 (98.3) | 12 (1.7) | 697 (98.9) | 8 (1.1) |
| | **Undergraduate** | 420 (96.3) | 16 (3.7) | 433 (99.3) | 3 (0.7) | 429 (98.4) | 7 (1.6) | 424 (97.2) | 12 (2.8) | 433 (99.3) | 3 (0.7) |
| | **University graduate** | 885 (97.7) | 21 (2.3) **NS** | 902 (99.4) | 5 (0.6) **NS** | 895 (98.7) | 12 (1.3) **NS** | 895 (98.7) | 12 (1.3) **NS** | 896 (98.8) | 11 (1.2) **NS** |
| **Father's level of education** | **No education** | 128 (97.0) | 4 (3.0) | 130 (98.5) | 2 (1.5) | 129 (97.7) | 3 (2.3) | 130 (98.5) | 2 (1.5) | 131 (99.2) | 1 (0.8) |
| | **Primary** | 578 (97.6) | 14 (2.4) | 587 (99.2) | 5 (0.8) | 582 (98.3) | 10 (1.7) | 578 (97.6) | 14 (2.4) | 585 (98.8) | 7 (1.2) |
| | **Complementary** | 915 (96.0) | 38 (4.0) | 950 (99.5) | 5 (0.5) | 937 (98.1) | 18 (1.9) | 938 (98.2) | 17 (1.8) | 945 (99.0) | 10 (1.0) |
| | **Secondary** | 681 (97.0) | 21 (3.0) | 698 (99.3) | 5 (0.7) | 695 (98.9) | 8 (1.1) | 693 (98.6) | 10 (1.4) | 696 (99.0) | 7 (1.0) |
| | **Undergraduate** | 274 (96.8) | 9 (3.2) | 281 (99.3) | 2 (0.7) | 278 (98.2) | 5 (1.8) | 278 (98.2) | 5 (1.8) | 280 (98.9) | 3 (1.1) |
| | **University graduate** | 439 (96.9) | 14 (3.1) **NS** | 453 (100.0) | 0 (0.0) **NS** | 446 (98.5) | 7 (1.5) **NS** | 447 (98.7) | 6 (1.3) **NS** | 450 (99.3) | 3 (0.7) **NS** |
| **Other factors** | | | | | | | | | | | |
| **Medical history** | **No** | 1640 (96.8) | 54 (3.2) | 1685 (99.4) | 11 (0.6) | 1655 (97.6) | 41 (2.4) | 1660 (97.9) | 36 (2.1) | 1675 (98.8) | 21 (1.2) |
| | **Yes** | 1625 (96.5) | 59 (3.5) **NS** | 1671 (99.2) | 13 (0.8) **NS** | 1662 (98.7) | 22 (1.3) **p*** | 1654 (98.2) | 30 (1.8) **NS** | 1665 (98.9) | 19 (1.1) **NS** |
| **Ongoing treatment** | **No** | 2756 (96.9) | 88 (3.1) | 2825 (99.3) | 19 (0.7) | 2788 (98.0) | 56 (2.0) | 2789 (98.1) | 55 (1.9) | 2811 (98.8) | 33 (1.2) |
| | **Yes** | 473 (97.3) | 13 (2.7) **NS** | 487 (100.0) | 0 (0.0) **NS** | 477 (97.9) | 10 (2.1) **NS** | 479 (98.4) | 8 (1.6) **NS** | 481 (98.8) | 6 (1.2) **NS** |
| **Child visit to the pediatrician/doctor** | **No** | 652 (96.2) | 26 (3.8) | 667 (98.2) | 12 (1.8) | 660 (97.2) | 19 (2.8) | 663 (97.6) | 16 (2.4) | 670 (98.7) | 9 (1.3) |
| | **Yes** | 2543 (97.1) | 75 (2.9) **NS** | 2611 (99.7) | 7 (0.3) **p*** | 2571 (98.2) | 47 (1.8) **NS** | 2572 (98.2) | 46 (1.8) **NS** | 2589 (98.9) | 29 (1.1) **NS** |
| **Previous child visit to the psychologist** | **No** | 3091 (96.8) | 101 (3.2) | 3177 (99.4) | 19 (0.6) | 3138 (98.2) | 58 (1.8) | 3143 (98.3) | 53 (1.7) | 3163 (99.0) | 33 (1.0) |
| | **Yes** | 137 (95.8) | 6 (4.2) **NS** | 141 (98.6) | 2 (1.4) **NS** | 142 (99.3) | 1 (0.7) **NS** | 140 (97.9) | 3 (2.1) **NS** | 143 (100.0) | 0 (0.0) **NS** |

**p* = p < 0.05**

**NS = Non-significant p value > 0.05**

## Table S1C. Associations between referrals & sociodemographic data and other factors

| Referrals / Sociodemographic factors | | Referral for abnormal lung auscultation N (%) | | Referral for enlarged node or/and organ N (%) | | Referral for abnormal ear exam N (%) | | Referral for signs of early puberty N (%) | | Referral for abnormal neurological findings N (%) | |
|---|---|---|---|---|---|---|---|---|---|---|---|
| | | No | Yes | No | Yes | No | Yes | No | Yes | No | Yes |
| **Nationality** | **Lebanese** | 6601 (98.8) | 81 (1.2) | 6632 (99.3) | 50 (0.7) | 6383 (95.5) | 299 (4.5) | 6626 (99.3) | 49 (0.7) | 6638 (99.3) | 44 (0.7) |
| | **Syrian** | 478 (99.6) | 2 (0.4) | 480 (100.0) | 0 (0.0) | 467 (97.3) | 13 (2.7) | 474 (99.6) | 2 (0.4) | 479 (99.8) | 1 (0.2) |
| | **Palestinian** | 8 (100.0) | 0 (0.0) | 8 (100.0) | 0 (0.0) | 8 (100.0) | 0 (0.0) | 8 (100.0) | 0 (0.0) | 8 (100.0) | 0 (0.0) |
| | **Other** | 14 (100.0) | 0 (0.0) **NS** | 14 (100.0) | 0 (0.0) **NS** | 14 (100.0) | 0 (0.0) **NS** | 14 (100.0) | 0 (0.0) **NS** | 14 (100.0) | 0 (0.0) **NS** |
| **Gender** | **Male** | 3450 (98.8) | 41 (1.2) | 3462 (99.2) | 29 (0.8) | 3340 (95.7) | 151 (4.3) | 3452 (99.1) | 33 (0.9) | 3468 (99.3) | 23 (0.7) |
| | **Female** | 3651 (98.9) | 42 (1.1) **NS** | 3672 (99.4) | 21 (0.6) **NS** | 3532 (95.6) | 161 (4.4)**NS** | 3670 (99.5) | 18 (0.5) **p*** | 3671 (99.4) | 22 (0.6) **NS** |
| **Age** | **3-6 years** | 2602 (97.9) | 55 (2.1) | 2635 (99.2) | 22 (0.8) | 2501 (94.1) | 156 (5.9) | 2640 (99.4) | 17 (0.6) | 2635 (99.2) | 22 (0.8) |
| | **7-9 years** | 2288 (99.3) | 17 (0.7) | 2293 (99.5) | 12 (0.5) | 2238 (97.1) | 67 (2.9) | 2270 (98.5) | 34 (1.5) | 2294 (99.5) | 11 (0.5) |
| | **10-12 years** | 2211 (99.5) | 11 (0.5) **p*** | 2206 (99.3) | 16 (0.7) **NS** | 2133 (96.0) | 89 (4.0) **p*** | 2212 (100.0) | 0 (0.0) **p*** | 2210 (99.5) | 12 (0.5) **NS** |
| **BMI** | **Underweight** | 1913 (99.3) | 13 (0.7) | 1911 (99.2) | 15 (0.8) | 1853 (96.2) | 73 (3.8) | 1910 (99.2) | 15 (0.8) | 1918 (99.6) | 8 (0.4) |
| | **Normal weight** | 2827 (98.3) | 49 (1.7) | 2854 (99.2) | 22 (0.8) | 2739 (95.2) | 137 3(4.8) | 2846 (99.2) | 24 (0.8) | 2852 (99.2) | 24 (0.8) |
| | **Overweight** | 2361 (99.1) | 21 (0.9) **p*** | 2369 (99.5) | 13 (0.5) **NS** | 2280 (95.7) | 102 (4.3)**NS** | 2366 (99.5) | 12 (0.5) **NS** | 2369 (99.5) | 13 (0.5) **NS** |
| **Governorate** | **Beirut** | 291 (99.0) | 3 (1.0) | 291 (99.0) | 3 (1.0) | 258 (87.8) | 36 (12.2) | 288 (98.0) | 6 (2.0) | 292 (99.3) | 2 (0.7) |
| | **Mount Lebanon** | 170 (100.0) | 0 (0.0) | 170 (100.0) | 0 (0.0) | 167 (98.2) | 3 (1.8) | 167 (98.8) | 2 (1.2) | 168 (98.8) | 2 (1.2) |
| | **North/Akkar** | 1192 (98.9 | 13 (1.1) | 1202 (99.8) | 3 (0.2) | 1167 (96.8) | 38 (3.2) | 1197 (99.5) | 6 (0.5) | 1197 (99.3) | 8 (0.7) |
| | **Beqaa/Baalbek** | 3344 (98.9) | 38 (1.1) | 3343 (98.8) | 39 (1.2) | 3202 (94.7) | 180 (5.3) | 3348 (99.0) | 33 (1.0) | 3360 (99.3) | 22 (0.7) |
| | **South** | 2104 (98.6) | 29 (1.4) **NS** | 2128 (99.8) | 5 (0.2) **p*** | 2078 (97.4) | 55 (2.6) **p*** | 2122 (99.8) | 4 (0.2) **p*** | 2122 (99.5) | 11 (0.5) **NS** |
| **School type** | **Public** | 2427 (99.0) | 24 (1.0) | 2440 (99.6) | 11 (0.4) | 2382 (97.2) | 69 (2.8) | 2424 (99.3) | 17 (0.7) | 2436 (99.4) | 15 (0.6) |
| | **Private** | 4674 (98.8) | 59 (1.2) **NS** | 4694 (99.2) | 39 (0.8) **NS** | 4490 (94.9) | 243 (5.1) **p*** | 4698 (99.3) | 34 (0.7) **NS** | 4703 (99.4) | 30 (0.6) **NS** |
| **Family income** | **No income** | 273 (98.6) | 4 (1.4) | 275 (99.3) | 2 (0.7) | 261 (94.2) | 16 (5.8) | 275 (100.0) | 0 (0.0) | 275 (99.3) | 2 (0.7) |
| | **<100$** | 1214 (98.6) | 17 (1.4) | 1215 (98.7) | 16 (1.3) | 1165 (94.6) | 66 (5.4) | 1221 (99.3) | 8 (0.7) | 1224 (99.4) | 7 (0.6) |
| | **100-300$** | 328 (98.8) | 4 (1.2) | 329 (99.1) | 3 (0.9) | 316 (95.2) | 16 (4.8) | 331 (99.7) | 1 (0.3) | 327 (98.5) | 5 (1.5) |
| | **300-600$** | 894 (99.1) | 8 (0.9) | 901 (99.9) | 1 (0.1) | 859 (95.2) | 43 (4.8) | 887 (98.3) | 15 (1.7) | 898 (99.6) | 4 (0.4) |

| | | | | | | | | | | | | | | | | |
|---|---|---|---|---|---|---|---|---|---|---|---|---|---|---|---|---|
| | **600-900$** | 222 (97.8) | 5 (2.2) | | 223 (98.2) | 4 (1.8) | | 212 (93.4) | 15 (6.6) | | 225 (99.1) | 2 (0.9) | | 226 (99.6) | 1 (0.4) | |
| | **>900$** | 26 (96.3) | 1 (3.7) | **NS** | 27 (100.0) | 0 (0.0) | **p*** | 27 (100.0) | 0 (0.0) | **NS** | 27 (100.0) | 0 (0.0) | **p*** | 27 (100.0) | 0 (0.0) | **NS** |
| **Mother's occupation** | **No work** | 3 (100.0) | 0 (0.0) | | 3 (100.0) | 0 (0.0) | | 3 (100.0) | 0 (0.0) | | 3 (100.0) | 0 (0.0) | | 3 (100.0) | 0 (0.0) | |
| | **Housewife** | 2223 (98.8) | 1137 (50.5) | | 2234 (99.2) | 148 (6.6) | | 2138 (95.0) | 316 (14.0) | | 2232 (99.3 | 35 (1.6) | | 2239 (99.5 | 432 (19.2) | |
| | **Student** | 16 (100.0) | 4 (25.0) | | 16 (100.0) | 0 (0.0) | | 16 (100.0) | 3 (18.8) | | 16 (100.0) | 0 (0.0) | | 16 (100.0) | 1 (6.3) | |
| | **Employed** | 582 (98.8) | 297 (50.4) | | 584 (99.2) | 22 (3.7) | | 561 (95.2) | 98 (16.6) | | 582 (98.8) | 15 (2.5) | | 583 (99.0) | 95 (16.1) | |
| | **Self-employed** | 133 (99.3) | 66 (49.3) | | 134 (100.0) | 9 (6.7) | | 125 (93.3) | 17 (12.7) | | 133 (99.3) | 1 (0.7) | | 134 (100.0) | 23 (17.2) | |
| | **Unemployed** | 87 (98.9) | 44 (50.0) | | 87 (98.9) | 2 (2.3) | | 86 (97.7) | 11 (12.5) | | 86 (97.7) | 1 (1.1) | | 88 (100.0) | 11 (12.5) | |
| | **Retired** | 9 (100.0) | 0 (0.0) | | 9 (100.0) | 0 (0.0) | | 9 (100.0 | 0 (0.0) | | 9 (100.0) | 0 (0.0) | | 9 (100.0) | 0 (0.0) | |
| | **Disabled** | 2 (100.0) | 1 (50.0) | | 2 (100.0) | 0 (0.0) | | 2 (100.0) | 1 (50.0) | | 2 (100.0) | 0 (0.0) | | 2 (100.0) | 0 (0.0) | |
| | **Health field** | 74 (97.4) | 29 (38.2) | | 74 (97.4) | 1 (1.3) | | 74 (97.4) | 6 (7.9) | | 76 (100.0) | 0 (0.0) | | 76 (100.0) | 7 (9.2) | |
| | **Other** | 33 (100.0) | 19 (57.6) | **p*** | 33 (100.0) | 0 (0.0) | **NS** | 32 (97.0) | 4 (12.1) | **NS** | 33 (100.0) | 2 (6.1) | **NS** | 33 (100.0) | 7 (21.2) | **NS** |
| **Father's occupation** | **No work** | 8 (100.0) | 0 (0.0) | | 8 (100.0) | 0 (0.0) | | 8 (100.0) | 0 (0.0) | | 8 (100.0) | 0 (0.0) | | 8 (100.0) | 0 (0.0) | |
| | **Housewife** | 5 (100.0) | 0 (0.0) | | 5 (100.0) | 0 (0.0) | | 4 (80.0) | 1 (20.0) | | 5 (100.0) | 0 (0.0) | | 5 (100.0) | 0 (0.0) | |
| | **Student** | 11 (100.0) | 0 (0.0) | | 11 (100.0) | 0 (0.0) | | 11 (100.0) | 0 (0.0) | | 11 (100.0) | 0 (0.0) | | 11 (100.0) | 0 (0.0) | |
| | **Employed** | 1324 (98.4) | 21 (1.6) | | 1329 (98.8) | 16 (1.2) | | 1274 (94.7) | 71 (5.3) | | 1331 (99.0) | 14 (1.0) | | 1334 (99.2) | 11 (0.8) | |
| | **Self-employed** | 1097 (98.8) | 13 (1.2) | | 1103 (99.4) | 7 (0.6) | | 1053 (94.9) | 57 (5.1) | | 1098 (99.1) | 10 (0.9) | | 1107 (99.7) | 3 (0.3) | |
| | **Unemployed** | 211 (99.1) | 2 (0.9) | | 213 (100.0) | 0 (0.0) | | 203 (95.3) | 10 (4.7) | | 213 (100.0) | 0 (0.0) | | 212 (99.5) | 1 (0.5) | |
| | **Retired** | 118 (100.0) | 0 (0.0) | | 118 (100.0) | 0 (0.0) | | 113 (95.8) | 5 (4.2) | | 118 (100.0) | 0 (0.0) | | 116 (98.3) | 2 (1.7) | |
| | **Disabled** | 17 (100.0) | 0 (0.0) | | 17 (100.0) | 0 (0.0) | | 16 (94.1) | 1 (5.9) | | 16 (94.1) | 1 (5.9) | | 17 (100.0) | 0 (0.0) | |
| | **Health field** | 35 (100.0) | 0 (0.0) | | 35 (100.0) | 0 (0.0) | | 33 (94.3) | 2 (5.7) | | 34 (97.1) | 1 (2.9) | | 35 (100.0) | 0 (0.0) | |
| | **Other** | 231 (98.7) | 3 (1.3) | **NS** | 233 (99.6) | 1 (0.4) | **NS** | 227 (97.0) | 7 (3.0) | **NS** | 234 (100.0) | 0 (0.0) | **NS** | 234 (100.0) | 0 (0.0) | **NS** |
| **Mother's level of education** | **No education** | 73 (100.0% | 0 (0.0) | | 73 (100.0) | 0 (0.0) | | 69 (94.5) | 4 (5.5) | | 73 (100.0) | 0 (0.0) | | 73 (100.0) | 0 (0.0) | |
| | **Primary** | 397 (99.5% | 2 (0.5) | | 394 (98.7) | 5 (1.3) | | 376 (94.2) | 23 (5.8) | | 399 (100.0) | 0 (0.0 | | 397 (99.5) | 2 (0.5) | |
| | **Complementary** | 717 (99.3% | 5 (0.7) | | 717 (99.3) | 5 (0.7) | | 694 (96.1) | 28 (3.9) | | 717 (99.4) | 4 (0.6) | | 718 (99.4) | 4 (0.6) | |
| | **Secondary** | 696 (98.7% | 9 (1.3 | | 701 (99.4) | 4 (0.6) | | 676 (95.9) | 29 (4.1) | | 694 (98.7) | 9 (1.3) | | 701 (99.4) | 4 (0.6) | |
| | **Undergraduate** | 423 (97.0% | 13 (3.0) | | 431 (98.9) | 5 (1.1) | | 408 (93.6) | 28 (6.4) | | 431 (98.9) | 5 (1.1) | | 435 (99.8) | 1 (0.2) | |
| | **University graduate** | 897 (98.9% | 10 (1.1) | **p*** | 901 (99.3) | 6 (0.7) | **NS** | 863 (95.1) | 44 (4.9) | **NS** | 899 (99.1) | 8 (0.9) | **NS** | 899 (99.1) | 8 (0.9) | **NS** |
| **Father's level of education** | **No education** | 131 (99.2) | 1 (0.8) | | 132 (100.0 | 0 (0.0) | | 129 (97.7) | 3 (2.3) | | 131 (99.2) | 1 (0.8) | | 132 (100.0 | 0 (0.0) | |
| | **Primary** | 586 (99.0) | 6 (1.0) | | 587 (99.2) | 5 (0.8) | | 558 (94.3) | 34 (5.7) | | 585 (99.2) | 5 (0.8) | | 587 (99.2) | 5 (0.8) | |
| | **Complementary** | 941 (98.5) | 14 (1.5) | | 950 (99.5) | 5 (0.5) | | 912 (95.5) | 43 (4.5) | | 949 (99.4) | 6 (0.6) | | 951 (99.6) | 4 (0.4) | |
| | **Secondary** | 694 (98.7) | 9 (1.3) | | 699 (99.4) | 4 (0.6) | | 667 (94.9) | 36 (5.1) | | 698 (99.3) | 5 (0.7) | | 699 (99.4) | 4 (0.6) | |
| | **Undergraduate** | 275 (97.2) | 8 (2.8) | | 278 (98.2) | 5 (1.8) | | 269 (95.1) | 14 (4.9) | | 281 (99.3) | 2 (0.7) | | 281 (99.3) | 2 (0.7) | |
| | **University graduate** | 452 (99.8) | 1 (0.2) | **NS** | 448 (98.9) | 5 (1.1) | **NS** | 428 (94.5) | 25 (5.5) | **NS** | 446 (98.5) | 7 (1.5) | **NS** | 451 (99.6) | 2 (0.4) | **NS** |
| Other factors | | | | | | | | | | | | | | | | |
| **Medical history** | **No** | 1673 (98.6) | 23 (1.4) | | 1683 (99.2) | 13 (0.8) | | 1601 (94.4) | 95 (5.6) | | 1680 (99.2) | 14 (0.8) | | 1679 (99.0) | 17 (1.0) | |
| | **Yes** | 1665 (98.9) | 19 (1.1) | **NS** | 1672 (99.3) | 12 (0.7) | **NS** | 1621 (96.3) | 63 (3.7) | **p*** | 1668 (99.2) | 13 (0.8) | **NS** | 1674 (99.4) | 10 (0.6) | **NS** |
| **Ongoing treatment** | **No** | 2802 (98.5) | 42 (1.5) | | 2823 (99.3) | 21 (0.7) | | 2724 (95.8) | 120 (4.2) | | 2819 (99.4) | 18 (0.6) | | 2824 (99.3) | 20 (0.7) | |
| | **Yes** | 479 (98.4) | 8 (1.6) | **NS** | 480 (98.6) | 7 (1.4) | **NS** | 463 (95.1) | 24 (4.9) | **NS** | 483 (99.2) | 4 (0.8) | **NS** | 484 (99.4) | 3 (0.6) | **NS** |
| **Child visit to the pediatrician/doctor** | **No** | 667 (98.2) | 12 (1.8) | | 676 (99.6) | 3 (0.4) | | 645 (95.0) | 34 (5.0) | | 672 (99.4) | 4 (0.6) | | 674 (99.3) | 5 (0.7) | |
| | **Yes** | 2580 (98.5) | 38 (1.5) | **NS** | 2593 (99.0) | 25 (1.0) | **NS** | 2508 (95.8) | 110 (4.2) | **NS** | 2596 (99.3) | 18 (0.7) | **NS** | 2600 (99.3) | 18 (0.7) | **NS** |
| **Previous child visit to the psychologist** | **No** | 3155 (98.7) | 41 (1.3) | | 3170 (99.2) | 26 (0.8) | | 3042 (95.2) | 154 (4.8) | | 3169 (99.3) | 23 (0.7) | | 3179 (99.5) | 17 (0.5) | |
| | **Yes** | 143 (100.0) | 0 (0.0) | **NS** | 143 (100.0) | 0 (0.0) | **NS** | 134 (93.7) | 9 (6.3) | **NS** | 140 (97.9) | 3 (2.1) | **NS** | 140 (97.9) | 3 (2.1%) | **p*** |

**p* = p < 0.05**

**NS = Non-significant p value > 0.05**
